# Supplementary material for: Development of core outcome sets and core outcome measures for central visual impairment, visual field loss and ocular motility disorders due to stroke: a Delphi and consensus study
Source: BMJ Open. 2022 Mar 18;12(3):e056792. doi: 10.1136/bmjopen-2021-056792 (PMC8935181; doi:10.1136/bmjopen-2021-056792)
Supplement: Supplementary data [file bmjopen-2021-056792supp001.pdf]

COMET initiative registration

https://www.comet-initiative.org/Studies/Details/1420

Development of core outcome sets for vision screening and assessment in stroke: a Delphi and consensus study

OBJECTIVES: Visual impairment following stroke is common with a reported incidence of visual impairment in 60% of stroke survivors. Screening for visual impairment is neither routine nor standardised. This results in a health inequality where some stroke survivors receive comprehensive vision assessment to identify any existent visual problems while others receive no vision assessment leaving them with unmet needs from undiagnosed visual problems. The aim of this study was to define two core outcome sets (COS), one for vision screening and one for full visual assessment of stroke survivors. DESIGN: A list of potentially relevant visual assessments was created from a review of the literature. The consensus process consisted of an online 3-round Delphi survey followed by a consensus meeting of the key stakeholders. PARTICIPANTS: Stakeholders included orthoptists, occupational therapists, ophthalmologists, stroke survivors and COS users such as researchers, journal editors and guideline developers. SETTING: University. OUTCOME MEASURES: COS. RESULTS: Following the consensus process we recommend the following nine assessments for vision screening: case history, clinical observations of visual signs, visual acuity, eye alignment position, eye movement assessment, visual field assessment, visual neglect assessment, functional vision assessment and reading assessment. We recommend the following 11 assessments for full vision assessment: case history, observations, visual acuity, eye alignment position, eye movement assessment, binocular vision assessment, eye position measurement, visual field assessment, visual neglect assessment, functional vision assessment, reading assessment and quality of life questionnaires. CONCLUSIONS: COS are defined for vision screening and full vision assessment for stroke survivors. There is potential for their use in reducing heterogeneity in routine clinical practice and for improving standardisation and accuracy of vision assessment. Future research is required to evaluate the use of these COS and for further exploration of core outcome measures.

Aim

The aim of this study was to define two core outcome sets (COS), one for vision screening and one for full visual assessment of stroke survivors.

Contributors

Rowe, F. J. Hepworth, L. R. Kirkham, J. J.

Publication

Journal: BMJ Open
Volume: 9
Issue: 9
Pages: e029578 -
Year: 2019
DOI: 10.1136/bmjopen-2019-029578

Further Study Information

Current Stage: Not Applicable
Date:
Funding source(s): This article/paper/report presents independent research funded by the National Institute for Health Research (NIHR: CDF-2012-05-126).

Health Area

Disease Category: Eyes & vision, Neurology

Disease Name: Visual impairment after stroke

Target Population

Age Range: 18 - 100

Sex: Either

Nature of Intervention: Other, Screening

Stakeholders Involved

- Clinical experts
- Consumers (caregivers)
- Consumers (patients)
- Guideline developers
- Journal editors
- Researchers

Study Type

- COS for clinical trials or clinical research
- COS for practice

Method(s)

- Consensus meeting
- Delphi process
- Literature review

A list of potentially relevant visual assessments was created from a review of the literature. The consensus process consisted of an online 3-round Delphi survey followed by a consensus meeting of the key stakeholders.
