# Supplementary material for: Development of core outcome sets and core outcome measures for central visual impairment, visual field loss and ocular motility disorders due to stroke: a Delphi and consensus study
Source: BMJ Open. 2022 Mar 18;12(3):e056792. doi: 10.1136/bmjopen-2021-056792 (PMC8935181; doi:10.1136/bmjopen-2021-056792)
Supplement: Author's manuscript [file bmjopen-2021-056792.draft_revisions.pdf]

BMJ Open is committed to open peer review. As part of this commitment we make the peer review history of every article we publish publicly available.

When an article is published we post the peer reviewers' comments and the authors' responses online. We also post the versions of the paper that were used during peer review. These are the versions that the peer review comments apply to.

The versions of the paper that follow are the versions that were submitted during the peer review process. They are not the versions of record or the final published versions. They should not be cited or distributed as the published version of this manuscript.

BMJ Open is an open access journal and the full, final, typeset and author-corrected version of record of the manuscript is available on our site with no access controls, subscription charges or pay-per-view fees (http://bmjopen.bmj.com).

If you have any questions on BMJ Open's open peer review process please email info.bmjopen@bmj.com

BMJ Open

Development of core outcome sets and core outcome measures for central visual impairment, visual field loss and ocular motility disorders due to stroke: a Delphi and consensus study.

Journal:	*BMJ Open*
Manuscript ID	bmjopen-2021-056792
Article Type:	Original research
Date Submitted by the Author:	25-Aug-2021

Complete List of Authors:	Rowe, Fiona; University of Liverpool<br>Hepworth, Lauren; University of Liverpool, Health Services Research<br>Kirkham, Jamie J.; Manchester University, Biostatistics

Keywords:	Stroke < NEUROLOGY, STROKE MEDICINE, OPHTHALMOLOGY, Neuro-ophthalmology < OPHTHALMOLOGY

SCHOLARONE™
Manuscripts

I, the Submitting Author has the right to grant and does grant on behalf of all authors of the Work (as defined in the below author licence), an exclusive licence and/or a non-exclusive licence for contributions from authors who are: i) UK Crown employees; ii) where BMJ has agreed a CC-BY licence shall apply, and/or iii) in accordance with the terms applicable for US Federal Government officers or employees acting as part of their official duties; on a worldwide, perpetual, irrevocable, royalty-free basis to BMJ Publishing Group Ltd ("BMJ") its licensees and where the relevant Journal is co-owned by BMJ to the co-owners of the Journal, to publish the Work in this journal and any other BMJ products and to exploit all rights, as set out in our licence.

The Submitting Author accepts and understands that any supply made under these terms is made by BMJ to the Submitting Author unless you are acting as an employee on behalf of your employer or a postgraduate student of an affiliated institution which is paying any applicable article publishing charge ("APC") for Open Access articles. Where the Submitting Author wishes to make the Work available on an Open Access basis (and intends to pay the relevant APC), the terms of reuse of such Open Access shall be governed by a Creative Commons licence – details of these licences and which Creative Commons licence will apply to this Work are set out in our licence referred to above.

Other than as permitted in any relevant BMJ Author's Self Archiving Policies, I confirm this Work has not been accepted for publication elsewhere, is not being considered for publication elsewhere and does not duplicate material already published. I confirm all authors consent to publication of this Work and authorise the granting of this licence.

For peer review only

Development of core outcome sets and core outcome measures for central visual impairment, visual field loss and ocular motility disorders due to stroke: a Delphi and consensus study.

Fiona J Rowe[1], PhD, Lauren R Hepworth[1], PhD, Jamie J Kirkham[2], PhD

1, Institute of Population Health, University of Liverpool, UK

2, Centre for Biostatistics, University of Manchester, UK

Address for correspondence:

Prof Fiona Rowe

Waterhouse Building Block B,

University of Liverpool,

1-3 Brownlow Street,

Liverpool L69 3GL

T: 0151 7944956

E: rowef@liverpool.ac.uk

Twitter: @orthopticbrain

Category: Original research article

Short title: Core outcomes for vision and stroke

Word count: 4263

Number of tables: 3

Number of figures: 3

Supplementary data: 1

For peer review only

For peer review only - http://bmjopen.bmj.com/site/about/guidelines.xhtml

Abstract

Objectives: Reporting of research for stroke-related visual impairment is inconsistent. The aim of this study was to define three core outcome sets (COS) and related core outcome measurements (COM) for central visual impairment, visual field loss and ocular motility disorders in stroke research.

Design: The consensus process consisted of an online 3-round Delphi survey followed by a consensus meeting of the key stakeholders.

Setting: UK-wide survey.

Participants: Stakeholders included orthoptists, occupational therapists, ophthalmologists, stroke survivors and COS users such as researchers, journal editors and guideline developers.

Outcome measures: For COS development, a list of potentially relevant visual outcomes was created from a review of the literature and further grouped into outcome domains. For COM development, a list of potential instruments was created from a review of the literature and quality appraised for reliability and validity.

Results: Following the consensus process we recommend the following outcome domains for central visual impairment (visual acuity, functional vision, quality of life), visual field loss (visual fields, functional vision, quality of life) and ocular motility disorders (eye alignment, eye movements, functional vision, quality of life). Recommended instruments for measurement of these outcomes include; LogMAR visual acuity, cover test, cardinal position eye movement assessments, peripheral visual field perimetry, Visual Function Questionnaire-25.

Conclusions: COS and COM are defined for vision research for stroke survivors. There is potential for their use to reduce heterogeneity in routine clinical practice and to improve standardisation and accuracy of vision assessment. Future research is required to evaluate the use of these COS and COM.

Keywords: Core outcome set; Core outcome measures; Vision; Stroke; Delphi; Consensus; Central vision; Visual field loss; Ocular motility

For peer review only

Strengths and limitations of this study

- We report a new core outcome set (COS) and core outcome measures (COM) for stroke-vision research.
- The COS and COM have a break-down for category of visual impairment; specifically, central vision impairment, visual field loss and ocular motility disorders.
- Composite outcome domains were ranked by Delphi and consensus opinion from multi-disciplinary experts and stroke survivors.
- This was not an international study; participants were from UK and Ireland.
- We experienced attrition bias across the Delphi rounds.

Declarations of interest

Funding: This article/paper/report presents independent research funded by the National Institute for Health Research (NIHR: CDF-2012-05-126). The views expressed are those of the authors and not necessarily those of the NHS, the NIHR or the Department of Health.

Conflicts of interest: All authors declare: no financial relationships with any organisations that might have an interest in the submitted work in the previous three years; no other relationships or activities that could appear to have influenced the submitted work.

Data availability: The Delphi dataset is available from the lead author on reasonable request.

Ethics approval: This study had institutional ethical approval (Ref-1415-040) and was undertaken in accordance with the Tenets of Helsinki. Informed consent was obtained for the Delphi survey and from all participants in the consensus meeting.

Author contributions: FR provided oversight for the study and led the writing of the paper. FR, LH and JK contributed to data collection, reviewing the draft paper and approving the final version.

For peer review only

Introduction

Visual impairment is common as a consequence to stroke with a reported incidence of 60% in stroke survivors [1]. Types of visual impairment generally fall into one of four categories including impairments of central vision, eye movements, visual fields and visual perception [2]. The impact of visual impairment on stroke survivors is wide reaching. Visual impairment can increase risk of collisions and falls, impede activities of daily life such as reading and writing, prevent return to work, pre-stroke activities and hobbies, and driving [3-6]. Such impacts result in loss of independence, increase likelihood of social isolation and results in mood change, anxiety and depression [5,6]. There are several approaches to the management of these visual impairments including visual scanning therapy, prisms, occlusion, spectacles, drugs, surgery, botulinum toxin, exercises, or a combination of two or more of the above [7-9].

A range of systematic reviews in the field of stroke-vision research have identified that there is considerable variation in the outcomes being measured and reported in primary research studies, which impacts the ability to compare and synthesise outcome results across studies. Moreover, it was noted that there is a paucity of outcome data available on important patient outcomes such as quality of life and functional visual assessment [7-11].

To mitigate these issues and to increase the relevance of research, a core outcome set (COS) and core outcome measurements (COM) can be developed which represents an agreed standardised set of outcomes and instruments that should be used and reported in all studies for a specific area of health or healthcare [12,13]. A search of the COMET (Core Outcome Measures in Effectiveness Trials) database revealed that there are several studies that have investigated important outcomes for eyes and vision disease; examples include cataracts and glaucoma but none have specifically looked at stroke [14].

The aim of this study was to achieve consensus on the content of vision research outcomes for stroke survivors. In this study we report the results of a Delphi process and consensus meetings in the development of three COS and COM for stroke-

For peer review only - http://bmjopen.bmj.com/site/about/guidelines.xhtml

vision research for the categories of impaired central vision, visual field loss and ocular motility disorders.

Methods

Ethical approval

This study had institutional ethical approval (Ref-1415-040) and was undertaken in accordance with the Tenets of Helsinki. Informed consent was obtained for the Delphi survey from all participants in the consensus meeting.

Development of the COS involved three phases: (1) the generation of a comprehensive list of outcomes; (2) a Delphi survey involving three rounds to gain consensus as to which outcomes are most important; and (3) patient and professional consensus meetings to agree on a final COS.

Development of the COM involved three phases: (1) the generation of a comprehensive list of outcome measurement instruments specific to the outcomes in each COS, (2) quality appraisal of the outcome measurement instruments and (3) a consensus process on recommendations for the selection of outcome measurement instruments.

A protocol for the development of this COS project was written by the steering committee, registered in the COMET initiative website (http://www.comet-initiative.org/studies/details/275?result=true) and available as open access (http://pcwww.liv.ac.uk/~rowef/index_files/Page356.html). When developing this COS, we followed the minimum set of development standards set out by COS-STAD (Core Outcome Set-STAndards for Development) Statement and COSMIN (Consensus-based Standards for the selection of health Measurement Instruments), and report the results against the COS-STAR (Core Outcome Set–STAndards for Reporting) guideline [13,15,16].

The full methods for the COS have been fully outlined previously [17] and detail the steering group, patient and public involvement, and stakeholder groups.

Patient and public involvement

For peer review only - http://bmjopen.bmj.com/site/about/guidelines.xhtml

This study was supported by a patient advisory group (the VISable patient and public involvement panel) which provided input to this research study. The VISable panel met on a regular basis during the conduct of the study. Patients contributed to the design of the study and were involved at all stages of the survey and consensus meeting.

Core outcome set

Phase 1 – Outcome identification

A literature review was conducted to develop a preliminary list of outcomes for the Delphi survey. We undertook an overview of seven systematic reviews of studies/trials reporting vision screening, assessment and treatment of post-stroke visual impairment [2,7-11,18-20] and extracted a list of included outcomes. The list was then circulated to the VISable patient and public involvement panel [21] for approval and checking of writing for lay terms as the basis of the online survey development. VISable (a panel of ten patients/carers) were asked to consider whether any further outcomes should be added, particularly those deemed relevant to patient-reported outcomes. The panel advised that appropriate patient-important outcomes were included already in the list and did not add any further outcomes.

Phase 2 – Delphi survey

We undertook a prospective consensus study using a Delphi process. SurveyMonkey (SurveyMonkey Inc. Palo Alto, California, USA 2015) was used as the online platform to administer the Delphi process. The survey was piloted for checks of ambiguity and appropriate use of lay language by the steering group and then released live. The full survey was circulated through professional, clinical and research networks in the UK and Ireland to reach stakeholder groups of eye care teams (orthoptists, ophthalmologists), stroke teams (physicians, nurses, therapists) and patients (stroke survivors).

The Delphi survey consisted of three rounds. Rounds remained open for 10 weeks, with regular two-week reminders sent to those that had partially completed or not completed the survey in order to maximise response rates. All terms had explanatory

For peer review only - http://bmjopen.bmj.com/site/about/guidelines.xhtml

notes to aid interpretation. All participants were asked to score each outcome in terms of importance and asked to identify any additional outcomes of importance that did not appear in the list of assessments. Additional outcomes added in round 1 were reviewed by the steering group to consider their relevance to the survey and to identify and remove duplicates.

Scoring Method: In each round, participants were asked to score the importance of each outcome listed on a nine-point scale (1-3: not important; 4-6: important but not critical; 7-9: critical) as well as an ‘unable to score’ option. The scale was devised by the GRADE working group [22] to score the quality of evidence for outcomes in systematic reviews and has been adopted in other core outcome set development work research using Delphi methods.

Methods of analysis: For each round of Delphi, the results for each outcome were summarised, which included providing the number of participants responding to the survey, as well as the mean, standard deviation and the percentage of participants scoring the outcome at each possible level from 1-9.

In round 2, all data from round 1 was analysed and compiled by stakeholder groups;1) stroke survivors/carers, 2) stroke team professionals and 3) eye team professionals, to allow different perspectives to be considered prior to re-rating [12]. The summarised results for each of the three stakeholder groups were provided to all participants in the form of histogram plots. Each participant was also shown their personal original score for each outcome. Participants had the opportunity to re-score based on the summary scores from the three stakeholder groups versus their previous personal scores. All participants were also asked to score on additional outcomes that were identified and added in round 1.

In round 3, the results from round 2 were analysed. Following analysis, the results from each stakeholder group were reviewed by the steering group and judged to be similar in terms of mean responses and percentage spread across the responses of 1 to 9. Thus, they were presented as one compilation of all responses. Each participant was shown their personal score from round 2 for each outcome along with the summary scores from all other participants, and asked to score each outcome again in terms of importance.

Consensus was defined *‘a priori’* however, this information was not provided on the Delphi survey. Participants were aware of these cut off values at the time of

For peer review only - http://bmjopen.bmj.com/site/about/guidelines.xhtml

attending the consensus meeting and these values were applied overall to responses: ‘Consensus in’ (i.e. consensus that the outcome should be included in the core set) was defined as greater than 70% of participants scoring as 7-9 and less than 15% of participants scoring 1-3. ‘Consensus out’ (i.e. consensus that the outcome should not be included in the core set) was defined as greater than 70% of participants scoring as 1-3 and less than 15% of participants scoring as 7-9. All other combinations were seen as equivocal. The outcomes that were designated as ‘consensus in’ or seen as ‘equivocal’ were taken forward and discussed in more detail at the consensus meeting for inclusion into the final core outcome sets. Participants were reminded of all outcomes not reaching consensus as part of the Delphi process.

Phase 3 – Consensus meeting

All participants from the Delphi survey were invited to contribute to the consensus meeting. Those expressing interest were invited to attend a face to face consensus meeting. All round 3 survey completers were emailed an invitation. Our intention was to ensure all stakeholders were reasonably represented. The format of the meeting included a short study overview which outlined the categories of visual impairment due to stroke and outcomes identified from the outcome identification phase, a presentation containing a summary of the results and number of outcomes reaching consensus from the survey. Each outcome was be considered in turn, in order of their presentation in the Delphi survey, to ratify these results. Each remaining outcome was then be considered in turn with full discussion. Similar to the survey, each outcome was considered as reaching consensus with 70% of participants voting in favour of its inclusion.

Each participant voted on every outcome being asked to vote ‘Yes’ (this outcome should be included in the COS), ‘No’ (this outcome should not be included), or ‘Unsure’ using voting slips. Results were collated after voting for all outcomes was completed and results then presented to the group. Outcomes were retained or dropped when consensus is reached. Discussion and further rounds of voting, restricting the options to ‘Yes’ or ‘No’ was undertaken until consensus was reached on all outcomes. All outcomes retained were to be included in the final COS.

For peer review only

For peer review only - http://bmjopen.bmj.com/site/about/guidelines.xhtml

Core outcome measures

Phase 1 – Outcome identification

From the same literature review as the COS, a comprehensive list of outcome measurement instruments was generated, with circulation of the list to the VISable panel for review.

Phase 2 – Quality appraisal

We undertook a quality appraisal of the available outcome measurements identified from the literature review. For each outcome measurement instrument, we conducted a further review of the medical literature to seek evidence of the use of the instrument in a stroke population. We evaluated the validity of the instrument and feasibility for its use with stroke survivors as per COSMIN guidance [13]. Measurement properties included content validity, internal structure, reliability, measurement error, hypotheses testing, cross-culture validity, criterion validity and responsiveness. Feasibility aspects included comprehensibility, interpretability, ease and type of administration, length of the instrument, completion time, patient mental and physical ability level, ease of standardisation and scoring, type of instrument, cost, equipment required, availability in different settings, copyright, and regulatory approval requirements. Quality of evidence was graded from low to high.

Phase 3 – Consensus meeting

A subsequent (and separate to COS meeting) consensus meeting was planned for discussion of the COM development. Representatives from the COS Delphi survey were invited to a face to face consensus meeting; all round 3 survey completers were emailed an invitation. The intention was to ensure all stakeholders were reasonably represented. The same process as outlined above for COS development was followed for introduction to the process, definition of consensus, voting, and compilation of results. The plan was to seek consensus agreement on the inclusion of one instrument for each category of stroke-visual impairment research; specifically, categories of impaired central vision, ocular motility disorders and visual field loss. Consensus agreement was only sought for the agreed COS outcomes. Further, consensus agreement was sought on the timing of measurement.

For peer review only - http://bmjopen.bmj.com/site/about/guidelines.xhtml

Results

Figure 1 outlines a flowchart of the results for number of participants and number of outcomes in the development and agreement of the COS and COM.

Core outcome set process

Phase 1 - Outcome identification

We extracted 119 outcomes, many of which were variations on assessments for specific visual functions. For the purposes of clarity during COS development, similar assessments were also combined into outcome domains. This process produced a list of 24 domains (Table 1).

Phase 2 – Delphi survey

In total 123 participants registered for round 1 of the Delphi survey. Six registered but did not answer any survey questions. The remaining participants comprised of 79 orthoptists, 20 occupational therapists (stroke team stakeholder group), 17 stroke survivors (stroke survivor/carer stakeholder group) and 1 ophthalmologist. The orthoptists and ophthalmologist formed the eye team stakeholder group. There were 20 males and 97 females with ages ranging from 18-84 years. In round 2, 65 participants completed the survey – an attrition of 44.5% from round 1. These participants comprised of 47 orthoptists, 10 occupational therapists, 7 stroke survivors and 1 ophthalmologist. In round 3, 51 participants completed the survey – an attrition of 56.4% from round 1 (21.5% from round 2). These participants comprised of 39 orthoptists, 6 occupational therapists, 5 stroke survivors and 1 ophthalmologist.

Following completion of the survey of 119 outcomes in round 1, five additional outcomes were put forward by the participants for inclusion in round 2: red eye, eye contact, reaction to confrontation, extinction and voluntary/command saccades. Consensus ‘in’ scores were achieved for 72 outcomes. No outcomes scored less than 3. After round 2 of 124 outcomes, no new outcomes were introduced. Consensus ‘in’ scores were achieved for 67 outcomes. No outcomes scored less

For peer review only - http://bmjopen.bmj.com/site/about/guidelines.xhtml

than 3. Following round 3 of 124 outcomes, consensus ‘in’ scores were achieved for 75 outcomes. The Delphi survey reached the following consensus:

- Full consensus for inclusion was obtained for case history asking patients/carers open and specific questions plus checks of previous ocular history and glasses wear. Full consensus for observations, tests of visual acuity, eye alignment, eye movements, binocular vision, alignment measurement, visual fields, visual inattention, reading, lids and pupils, visual perception and functional visual assessment.
- No consensus was reached for eye contact during conversations, use of questionnaires, contrast sensitivity, colour vision, fundus checks and use of retinal photography/optical coherence tomography.

Phase 3 – Consensus meeting

The consensus meeting was a 1-day event held in Liverpool, UK with 12 participants comprising 5 occupational therapists (2 with research roles), 3 orthoptists (1 with a research role), 2 patients, 1 Cochrane editor and 1 facilitator. The facilitator did not take part in the voting. The objective of the meeting was to discuss and vote on the Delphi outcomes – the results of the Delphi survey had been provided to participants prior to and during the meeting.

During the consensus meeting, final consensus for research vision assessment was split according to category of visual impairment, (impaired central vision, visual field loss and ocular motility disorders). Participants decided to organise the COS on outcome domains rather than specific outcomes on the basis that many outcomes were similar and were better represented as a grouped outcome domain (see grouping of outcomes in Table 1). Two outcome domains were combined because of overlap in outcomes: functional vision and reading assessments.

No consensus could be reached for visual inattention or for visual perceptual disorders. Visual perceptual disorders were considered too heterogenous with so many different types of visual perception issue occuring. It was therefore not possible to determine a core set of assessments. Visual inattention was considered too broad and variable a condition with a discussion amongst consensus meeting participants about context and timing of assessment for stroke survivors with visual

For peer review only - http://bmjopen.bmj.com/site/about/guidelines.xhtml

attention. Agreement was therefore obtained only for categories of impaired central vision, visual field loss and eye movement disorders (Figure 2). Impaired central vision consisted of visual acuity, functional vision and quality of life. Visual field loss consisted of visual field perimetry, functional vision and quality of life. Eye movement disorders consisted of ocular alignment, ocular movement, functional vision and quality of life.

Core outcome measures process

Phase 1 - Outcome identification

From 119 outcomes identified for the COS, we extracted 52 test options. For the purposes of clarity, similar tests were combined into outcome domains. This process produced a list of 16 domains (Table 1).

Phase 2 – Quality appraisal

Quality assessment was conducted for the available outcome measurement instruments considering their measurement properties and feasibility aspects. Table 2 outlines the appraisal summary for selected instruments meeting quality indicators specific to the outcomes selected for the three COS of central vision impairment, visual field loss and ocular motility disorders; Logarithm of the Minimal Angle of Resolution (LogMAR) visual acuity, peripheral perimetry, prism cover test, ocular rotation grading, vision-related quality of life and reading.

Phase 3 – Consensus meeting

The consensus meeting was held as an online event with 13 participants comprising 2 occupational therapists, 7 orthoptists (2 with a research role), 3 patients and 1 facilitator. The facilitator did not take part in the voting. The objective of the meeting was to discuss and vote on the COM – the results of a comprehensive list of outcome measurements and their quality appraisal had been provided to participants prior to and during the meeting. Each participant was invited to make queries for each instrument and seek clarification of any part of the quality appraisal. Agreement was requested from each participant on inclusion of each selected instrument.

During the consensus meeting, final consensus for research vision assessment was split according to type of measurement (Figure 3). Overall excluded instruments and

For peer review only - http://bmjopen.bmj.com/site/about/guidelines.xhtml

reasons for exclusion are outlined in table 3. Recommendation for inclusion was obtained for;

- LogMAR test (impaired central vision). This test may require the use of a matching card because of communication or cognitive issues.
- Peripheral perimetry (visual field loss). Specifically, peripheral visual field testing was recommended over central visual field testing to acknowledge the need to detect presence of peripheral visual field loss outside the central 30 degrees and detection of change occurring in the peripheral visual field over time. Kinetic perimetry was recommended with the additional of static points to check the integrity of the visual field within the peripheral boundary. Where static-only programmes were available, a recommendation was made to use the Esterman programme which is most commonly available regardless of equipment type. A recommendation was made to use binocular Esterman programme as the minimum core assessment but with the addition of monocular Esterman fields for each eye where possible dependent on patient ability.
- Prism cover test and ocular rotation grading (ocular movement disorders). Ocular alignment determined by the cover test and measured by prism cover test. Ocular rotation gradings specifically in nine positions of gaze and assessed using saccadic and smooth pursuit movements alongside vergence (convergence and divergence). Recommendation to incorporate vestibulo-ocular reflex movements and/or optokinetic nystagmus where relevant (e.g. if smooth pursuit movements cannot be fully examined).
- VFQ-25 questionnaire (vision-related quality of life). Recommendation to add neuro-10 supplement when used for ocular motility COS but not appropriate for use in impaired central vision or visual field loss COS.
- Radner test (functional vision: reading impairment). Recommendation to capture additional impact to reading by non-visual issues such as presence of aphasia.

No consensus could be reached for functional vision assessment incorporating activities of daily living. Functional visual assessment was considered too heterogenous for types of assessment in relation to visual function versus general

For peer review only - http://bmjopen.bmj.com/site/about/guidelines.xhtml

function and therefore it was not possible to determine one, or even a small number, of measurement tools. Consensus was reached that the aspect of functional vision with respect to activities of daily living warrants further research and should consider the issues identified during the Delphi process and focus groups, including; observed navigation, eye scanning, walking observations, activities of daily living, self-care, body placement, spatial awareness, mobility observations, writing, hand-eye coordination, visual memory and cognition and visual perception.

The final stage of the consensus meeting was to consider the time points at which the COM should be made as a minimum. Consensus was achieved for a recommended measurement timescale of baseline and follow-up periods of 4 weeks, 12 weeks and 26 weeks, for all three COS. There was a recommendation that consideration should be given to recruitment in acute versus chronic stages of stroke given that rapid changes may occur in visual function within the first month of stroke onset.

Discussion

A core outcome set (COS) is an agreed minimum set of outcome measures that should be reported. By reporting a minimum set of measures, this reduces the heterogeneity of outcomes across studies, which in turn supports future evidence synthesis [12,15,16]. Once a COS is defined, it is important to achieve consensus on how the outcomes should be measured [13]. Core outcome measurements (COM) define how to measure core outcomes and are the instruments selected to measure each core outcome [13].

We have previously used Delphi and consensus process methods to develop test batteries for general clinical vision screening and assessment for post-stroke visual impairment [17]. The purpose of this present study was to agree on a COS and COM specific to use in stroke-vision research, distinct from routine clinical screening and assessment practice. After round 3 and the consensus meeting, stakeholders agreed with the proposed COS for vision research for three categories of visual impairment, consisting of three outcome domains for impaired central vision, three for visual field loss and four for eye movement disorders. Core amongst all categories were the

For peer review only - http://bmjopen.bmj.com/site/about/guidelines.xhtml

For peer review only

domains of functional vision and quality of life. It is important to acknowledge these COS do not exclude further visual assessments which should be added as appropriate and relevant for the individual research study.

Generally, one instrument is selected for each COM [13]. For assessment of impaired central vision, the selected instrument to test visual acuity was a logMAR chart, for visual field loss, peripheral perimetry was selected and for eye movement disorders, the selected instrument was the prism cover test when measuring ocular alignment, and grading of ocular rotations for measuring eye movement. For assessment of vision-related quality of life, the VFQ-25 questionnaire was selected and for functional vision assessment specific to reading, the Radner reading test was selected. It was not possible to reach consensus for measurement of other aspects of functional vision. There was unanimous agreement that further research is required to evaluate measurements of activities of daily living in relation to vision versus general function with specific aspects to be considered such as observed navigation and mobility, spatial awareness, self-care and writing. Quality appraisal for COM considered documentation of the use of the instrument in the target population, content validity, quality of evidence, and feasibility of use of the instrument [23-48]. Timing of these measurements were recommended at minimum time points of baseline and follow-up at 4, 12 and 26 weeks.

There are a number of strengths for this study. A COS has been produced for stroke-vision research with a break-down for category of visual impairment; specifically, central vision impairment, visual field loss and ocular motility disorders. This addresses a gap in evidence-based practice for post-stroke visual impairment. The COS is composed of outcome domains which were ranked by Delphi and consensus opinion from multi-disciplinary experts and stroke survivors. Furthermore, they are based on existent outcomes for which there are assessment instruments (i.e. COM) that are easily accessible for implementation, and accepted as validated clinical measures. Although these COS and COM were developed for stroke, there is potential for their use with other types of acquired brain injury causing visual impairment.

There are limitations to this study. Despite circulation of the survey across a wide range of networks, not all members of eye care and stroke teams took part, e.g. lack

For peer review only - http://bmjopen.bmj.com/site/about/guidelines.xhtml

of optometry, neuropsychology input. Participants were from UK and Ireland and largely represented views and practices within the National Health Service (NHS). Thus, assessments are those used clinically in these countries. However, we are aware that the assessment outcome domains selected for the COS and instruments selected for the COM are those that are widely used internationally in clinical and research settings as evidenced from our initial literature review [2,8,10]. Indeed, the quality appraisal of outcome measurement instruments involved many studies undertaken in countries other than the UK [23-48]. We experienced attrition bias across the Delphi rounds. The attrition rate at round 2 was 44.5% and, by round 3, was 56.4% from the initial sample. High attrition rates however are common using Delphi methods and our rates can be considered within an acceptable range based on those reported for other COS developments [49]. Nevertheless a larger sample would have been preferable. Researcher bias is also a potential limitation. We aimed to limit this by providing a summary of results across all rounds of the Delphi survey and with final decisions left to the consensus meeting with experts. There can be risks from using Delphi methods in which participants can have very disparate views of each outcome. However, we sought a wide variety of participants across a number of stakeholder groups to achieve greater consistency in responses and balance potential outlier responses. Further, this core outcome development process included a final stage of consensus meetings such that decisions were not purely made from the Delphi responses. Although we initially included outcomes for visual neglect/inattention and visual perception, it was not possible to reach consensus for a COS for these outcomes through the Delphi survey or consensus process. A detailed discussion took place during the consensus process that highlighted the specific issues for these outcomes and, in particular, the issue of context of assessment (where, when) and type (personal/peri-personal/extra-personal) of visual neglect, and significance of heterogeneity for forms of other visual perceptual disorders. Further it was not possible to reach consensus for a COM for functional vision specific to activities of daily living. Discussion during the consensus meeting highlighted that no specific instrument/tool for activities of daily living focussed on functional vision and that this represented a gap in evidence requiring further research. Whilst visual inattention and visual perception disorders were not included in the COS and COM developed in this study, these are topics that warrant further research to target core outcomes and measurements.

For peer review only - http://bmjopen.bmj.com/site/about/guidelines.xhtml

Conclusions

This study reports the use of Delphi and consensus methods in the development of a core outcome set and core outcome measurements for stroke-vision research. Impaired central vision and visual field loss each comprised three outcome domains and eye movement disorders comprised four outcome domains. Each COS outcome domain has one recommended outcome measurement instrument except for functional vision relating to activities of daily living for which no consensus could be reached. These core outcome sets and core outcome measurements will facilitate standardisation of stroke-vision assessment in order to reduce heterogeneity in assessment in future research. Further research is now required to evaluate the use of these core outcome sets and outcome measures.

Acknowledgements

We thank all participants involved in the Delphi survey and consensus meetings. We also thank our clinical and patient colleagues who contributed to the study steering group.

References

1. Rowe FJ, Hepworth LR, Howard C, Hanna KL, Cheyne CP, Currie J (2019) High incidence and prevalence of visual problems after acute stroke: An epidemiology study with implications for service delivery. PLoS ONE 14(3): e0213035. https://doi.org/10.1371/journal.pone.0213035
2. Hepworth LR, Rowe FJ, Walker MF, Rockliffe J, Noonan C, Howard C, Currie J. Post-stroke Visual Impairment: A Systematic Literature Review of Types and Recovery of Visual Conditions. Ophthalmology Research: An International Journal. 2015; 5(1). ISSN: 2321-7227
3. Hepworth L, Rowe FJ. Visual impairment following stroke – the impact on quality of life: a systematic review. Ophthalmology Research: an international journal. 2016; 5(2): 1-15

For peer review only - http://bmjopen.bmj.com/site/about/guidelines.xhtml

4. Jones SA, Shinton RA. Improving outcome in stroke patients with visual problems. Age Ageing. 2006; 35:560-5.
5. Tsai SY, Cheng CY, Hsu WM, Su TP, Liu JH, Chou P. Association between visual impairment and depression in the elderly. Journal of the Formosan Medical Association. 2003;102:86-90.
6. Chia E-M, Wang JJ, Rochtchina E, Smith W, Cumming RR, Mitchell P. Impact of bilateral visual impairment on health-related quality of life: The Blue Mountains eye study. Investigative Ophthalmology & Visual Science. 2004;45(1):71-76.
7. Pollock A, Hazelton C, Henderson CA, Angilley J, Dhillon B, Langhorne P, Livingstone K, Munro FA, Orr H, Rowe FJ, Shahani U. Interventions for visual field defects in patients with stroke. Cochrane Database of Systematic Reviews 2011, Issue 10. Art. No.: CD008388. DOI: 10.1002/14651858.CD008388.pub2.
8. Hanna KL, Hepworth LR, Rowe FJ. The treatment methods for post-stroke visual impairment: a systematic review. Brain and Behaviour. 2017; 7(5): e00682. DOI: 10.1002/brb3.682
9. Bowen A, Hazelton C, Pollock A, Lincoln NB. Cognitive rehabilitation for spatial neglect following stroke. Cochrane Database of Systematic Reviews 2013, Issue 7. Art. No.: CD003586. DOI: 10.1002/14651858.CD003586.pub3
10. Hanna KL, Hepworth R, Rowe FJ. Screening methods for post stroke visual impairment; a systematic review. Disability and Rehabilitation. 2016: DOI: 10.1080/09638288.2016.1231846
11. Hepworth L, Rowe FJ, Harper R, Jarvis K, Shipman T, Rodgers H. Patient reported outcome measures for visual impairment after stroke: a systematic review. Health and Quality of Life Outcomes. 2015; DOI: 10.1186/s12955-015-0338-x
12. Williamson PR, Altman DG, Bagley H, Barnes KL, Blazeby JM, Brookes ST, Clarke M, Gargon E, Gorst S, Harman N *et al*: The COMET Handbook: version 1.0. Trials 2017, 18(3):280.
13. Prinsen CAC, Vohra S, Rose MR, Boers M, Tugwell P, Clarke M, Williamson PR, Terwee CB. How to select outcome measurement instruments for outcomes included in a 'Core Outcome Set' – a practical guideline. Trials. 2016; 17: 449. DOI: 10.1186/s13063-016-1555-2

For peer review only - http://bmjopen.bmj.com/site/about/guidelines.xhtml

14. COMET-initiative. Core Outcome Measures in Effectiveness Trials. http://www.comet-initiative.org/ (last accessed 4th September 2020)
15. Kirkham JJ, Davis K, Altman DG, Blazeby JM, Clarke M, Tunis S, et al. (2017) Core Outcome Set-STAndards for Development: The COS-STAD recommendations. PLoS Med 14(11): e1002447. https://doi.org/10.1371/journal.pmed.1002447
16. Kirkham JJ, Gorst S, Altman DG, Blazeby JM, Clarke M, Devane D, et al. (2016) Core Outcome Set–STAndards for Reporting: The COS-STAR Statement. PLoS Med 13(10): e1002148. https://doi.org/10.1371/journal.pmed.1002148
17. Rowe FJ, Hepworth LR, Kirkham JJ. Development of core outcome sets for vision screening and assessment in stroke: a Delphi and consensus study. *BMJ Open* 2019;9:e029578. doi: 10.1136/bmjopen-2019-029578
18. Pollock A, Hazelton C, Henderson CA, Angilley J, Dhillon B, Langhorne P, Livingstone K, Munro FA, Orr H, Rowe FJ, Shahani U. Interventions for age-related visual problems in patients with stroke. Cochrane Database of Systematic Reviews 2012, Issue 3. Art. No.: CD008390. DOI: 10.1002/14651858.CD008390.pub2
19. Pollock A, Hazelton C, Henderson CA, Angilley J, Dhillon B, Langhorne P, Livingstone K, Munro FA, Orr H, Rowe FJ, Shahani U. Interventions for disorders of eye movement in patients with stroke. Cochrane Database of Systematic Reviews 2011, Issue 10. Art. No.: CD008389. DOI: 10.1002/14651858.CD008389.pub2.
20. Hanna KL, Rowe FJ. Clinical versus evidence-based rehabilitation options for post-stroke visual impairment. Neuro-Ophthalmology. 2017; epub. http://dx.doi.org/10.1080/01658107.2017.1337159
21. The Vision and Stroke patient and public group (VISable). www.vision-research.co.uk (last accessed 4th September 2020)
22. www.gradeworkinggroup.org (last accessed 4th September 2020)
23. Rowe FJ, VIS Group. Vision in Stroke cohort: profile overview of visual impairment. Brain and Behaviour. 2017*;e00771.* https://doi.org/10.1002/brb3.771

For peer review only - http://bmjopen.bmj.com/site/about/guidelines.xhtml

24. Rowe FJ, Conroy EJ, Bedson E, Cwiklinski E, Drummond A, García- Fiñana M, Howard C, Pollock A, Shipman T, Dodridge C, MacIntosh C, Johnson S, Noonan C, Barton G, Sackley C. A pilot randomised controlled trial comparing effectiveness of prism glasses, visual search training and standard care in hemianopia. Acta Neurologica Scandinavica. 2016; epub: DOI: 10.1111/ane.12725
25. Manny RE, Hussein M, Gwiazda J, Marsh-Tootle W, COMET study group. Repeatability of ETDRS visual acuity in children. Investigative Ophthalmology and Visual Science. 2003; 44: 3294-300
26. Stewart CE. LogMAR visual acuity measurements: limits of normality. British and Irish Orthoptic Journal. 2006; 3: 9-13
27. Negiloni K, Mazumdar D, Neog A, et al. Construction and validation of logMAR visual acuity charts in seven Indian languages. *Indian J Ophthalmol*. 2018;66(5):641-646. doi:10.4103/ijo.IJO_1165_17
28. Lam AK, Tong C, Tse J, Yu M. Repeatability of near visual acuity measurement at high and low contrast. *Clin Exp Optom*. 2008;91(5):447-452. doi:10.1111/j.1444-0938.2007.00235.x
29. Ferris FL 3rd, Bailey I. Standardizing the Measurement of Visual Acuity for Clinical Research Studies: Guidelines From the Eye Care Technology Forum. Ophthalmology. 1996 Jan;103(1):181-2.
30. Lempert P. Standardized Measurements of Visual Acuity for Clinical Research. Ophthalmology. 1996 Jul;103(7):994-5.
31. Ricci F, Cedrone C, Cerulli L. Standardized measurement of visual acuity. Ophthalmic Epidemiol. 1998 Mar;5(1):41-53.
32. Hull S, Tailor V, Balduzzi S, Rahi J, Schmucker C, Virgili G, Dahlmann-Noor A. Tests for detecting strabismus in children aged 1 to 6 years in the community. Cochrane Database of Systematic Reviews 2017, Issue 11. Art. No.: CD011221. DOI: 10.1002/14651858.CD011221.pub2.
33. Rowe FJ, Noonan CP, Manuel M. Comparison of Octopus Semi-Automated Kinetic Perimetry and Humphrey Peripheral Static Perimetry in Neuro-Ophthalmic Cases. ISRN Ophthalmology, vol. 2013, Article ID 753202. doi:10.1155/2013/753202

For peer review only - http://bmjopen.bmj.com/site/about/guidelines.xhtml

34. Rowe FJ, Rowlands A. Comparison of diagnostic accuracy between Octopus 900 and Goldmann kinetic visual fields. BioMed Research International. Volume 2014, Article ID 214829, doi: 10.1155/2014/214829
35. Ramirez AM, Chaya CJ, Gordon LK, Giaconi JA. (2008) A comparison of semiautomated versus manual Goldmann kinetic perimetry in patients with visually significant glaucoma. Journal of Glaucoma. 17: 111-117
36. Johnson CA. Standardizing the Measurement of Visual Fields for Clinical Research: Guidelines From the Eye Care Technology Forum. Ophthalmology. 1996 Jan;103(1):186-9.
37. Imaging and Perimetry Society Standards and Guidelines. Optom Vis Sci. 2011 Jan;88(1):4-7.
38. Kerr NM, Chew SS, Eady EK, Gamble GD, Danesh-Meyer HV. Diagnostic accuracy of confrontation visual field tests. Neurology. 2010 Apr 13;74(15):1184-90.
39. Holmes JM, Leske DA Hohberger GG. Defining Real Change in Prism-Cover Test Measurements. Am J Ophthalmol. 2008 Feb; 145(2): 381–385.
40. de Jongh E, Leach C, Tjon-Fo-Sang MJ, Bjerre A. Inter-examiner Variability and Agreement of the Alternate Prism Cover Test (APCT) Measurements of Strabismus Performed by 4 Examiners. Strabismus. 2014 Dec;22(4):158-66.
41. Rowe FJ. Ocular alignment and motility: moving variables in clinical trials. Trans. 34th European Strabismological Association. Bruges, Belgium. 2011, pp. 111-114
42. Rowe FJ. Measurement of manifest deviations using corneal reflections. Optometry Today, 1994 ; Nov : 36-38
43. Chen HF, Wu CY, Lin KC, Chen CL, Huang PC, Hsieh CJ, Liu JS. Rasch validation of a combined measure of basic and extended daily life functioning after stroke. Neurorehabilitation and Neural Repair. 2013; 27: 125-32
44. Radner W. Reading charts in ophthalmology/ Graefe's Archives Clinical Experimental Ophthalmology. 2017; 255: 1465-1482
45. Trauzettel-Klosinski S, Dietz K, IReST Steering Group. Standardised assessment of reading performance: the New International Reading peed Texts IReST. Investigative Ophthalmology and Vision Science. 2012; 53: 5452-61

For peer review only

46. Sarker SJ, Rudd AG, Douiri A, Wolfe CD. Comparison of 2 extended activities of daily living scales with the Barthel Index and predictors of their outcomes: cohort study within the South London Stroke Register (SLSR). Stroke. 2012; 43: 1362-9
47. Green J, Forster A, Young J. A test-retest reliability study of the Barthel Index, the Rivermead Mobility Index, the Nottingham Extended Activities of Daily Living Scale and the Frenchay Activities Index in stroke patients. Disability and Rehabilitation. 2001; 23: 670-6
48. Legg LA, Lewis SR, Schofield-Robinson OJ, Drummond A, Langhorne P. Occupational therapy for adults with problems in activities of daily living after stroke. Cochrane Database of Systematic Reviews 2017, Issue 7. Art. No.: CD003585. DOI: 10.1002/14651858.CD003585.pub3.
49. McKenna HP. The Delphi technique: a worthwhile research approach for nursing? Journal of Advanced Nursing. 1994; 19: 1221-25

Figures

Figure 1: Flow chart of COS and COM development process

Figure 2: Core outcome sets consensus

Figure 3: Core outcome measures consensus

For peer review only

For peer review only - http://bmjopen.bmj.com/site/about/guidelines.xhtml

Table 1, COS outcome extraction from overview of reviews

COM outcomes indicated in shaded cells

Outcomes for COS	COS domains
Case history – open questions	Case history – open questions
Case history – specific questions<br>Eye strain<br>Reading difficulty<br>Blurred, altered or reduced vision<br>Visual field loss<br>Awareness of full environment<br>Oscillopsia<br>Diplopia<br>Polyopia<br>Visual hallucinations<br>Altered colour vision<br>Altered movement of objects<br>Depth perception misjudgements<br>Tilted images<br>Distorted images<br>Face/object recognition<br>Clutter difficulty<br>Getting lost<br>Prolongation of images<br>Reverse image size<br>Glare<br>Visual crowding<br>Visual disorientation	Case history – specific questions
Case history – carer open questions	Case history – carer open questions
Case history – carer specific questions<br>Personal care issues<br>Eyes constantly moving/jerking<br>Missing things to one side<br>Bumping into things<br>Concerns over vision<br>Visual hallucinations<br>Family/friend recognition<br>Difficulty naming objects<br>Getting lost<br>Reading problems	Case history – carer specific questions
Case history – previous ocular history	Case history – previous ocular history
Case history – glasses wear	Case history – glasses wear

For peer review only - http://bmjopen.bmj.com/site/about/guidelines.xhtml

Observations – open comments	Observations – open comments
Observations – specific features<br>Lids<br>Pupils<br>Squint – misaligned eyes<br>Eye movements<br>Turning head to see<br>Closing one eye to see better<br>Misjudging distances<br>Wobbling eyes	Observations – specific features
logMAR charts<br>Snellen charts<br>Fixation and following observation<br>Vanishing optotype charts<br>Grating charts<br>Near acuity charts<br>Kay's pictures<br>Sheridan Gardiner single optotypes<br>Lea symbols	Visual acuity
Fundus check<br>Retinal photography/OCT	Ocular health
Cover uncover test<br>Alternating cover test<br>Observations of corneal reflections	Eye alignment position
Nine positions of gaze<br>Horizontal gaze only<br>Vertical gaze only<br>Horizontal and vertical gaze only<br>Vergence<br>Saccade movement<br>Smooth pursuit movement<br>Optokinetic nystagmus movement<br>Vestibulo-ocular reflex<br>Hess/Lees/Harms wall charts	Eye movement assessment
Retinal correspondence<br>Sensory fusion<br>Motor fusion<br>Stereopsis	Binocular vision assessment
Prism cover test<br>Krimsky test<br>Prism reflection test<br>Synoptophore<br>Bruckner test	Eye alignment measurements

For peer review only - http://bmjopen.bmj.com/site/about/guidelines.xhtml

Maddox rod	
Confrontation<br>Static central perimetry<br>Static peripheral perimetry<br>Kinetic perimetry	Visual field assessment
Line bisection<br>Cancellation task – star, balloon, heart,etc.<br>Clock drawing<br>Room/environment description<br>Behaviour inattention test battery	Visual neglect assessment
Observed navigation<br>Reading<br>Eye scanning<br>Walking observations<br>Activities of daily living<br>Self-care<br>Body placement<br>Spatial awareness<br>Mobility observations<br>Writing<br>Hand-eye coordination<br>Visual memory and cognition<br>Visual perception	Functional assessment
Special test, e.g. Wilkins, iRest, Radner<br>Newspaper, magazine<br>Book	Reading assessment
Vision-related, e.g. VFQ25, DLDV<br>Health-related, e.g. SF12<br>Activity of daily living, e.g. IADL<br>Extended activity of daily living, e.g. NEADL	Questionnaires
Swinging flashlight test	Pupil assessment
Palpebral apertures<br>Lid function test	Lid assessment
Pelli-Robson chart<br>Mars test<br>VisTech	Contrast sensitivity assessment
Ishihara test<br>City test	Colour vision assessment

OCT – Optical Coherence Tomography; iReST - International Reading Speed Test; VFQ25 – Visual Function Questionnaire 25; DLDV – Daily Living tasks Dependent

For peer review only - http://bmjopen.bmj.com/site/about/guidelines.xhtml

on Vision; SF12 – Short Form 12; IADL – Instrumental Activities of Daily Living; NEADL – Nottingham Extended Activities of Daily Living

For peer review only

For peer review only - http://bmjopen.bmj.com/site/about/guidelines.xhtml

Table 2, Quality indicators of included instruments

	**Visual acuity**	**Visual field**	**Ocular alignment**	**Ocular movement**	**Quality of life**	**Functional vision**
	LogMAR	***Peripheral perimetry***	***Prism cover test***	***Rotation grading***	***VFQ-25***	***Radner reading***
Use in target population	Yes	Yes	Yes	Yes	Yes	Yes
**Content validity**						
Reliability	+	+	+	+	+	+
Responsiveness	+	+	+	+	+	+
Internal consistency	+	+	+	+	+	+
Structural validity	+	+	+	+	+	+
Measurement error	+	+	+	+	+	+
Hypothesis testing	+	+	+	+	+	+
Criterion validity	+	+	+	+	+	+
Cross-cultural validity	+	+	+	+	+	+
**Quality of evidence**	High	High	Moderate	Moderate	Moderate	Moderate
**Feasibility**						

For peer review only - http://bmjopen.bmj.com/site/about/guidelines.xhtml

Patient comprehension	Widely understood	Widely understood	Widely understood	Widely understood	Widely understood	Widely understood
Interpretability	Clear usage	Clear usage	Clear usage	Clear usage	Clear usage	Clear usage
Ease of administration	High	Relative	High	High	High	High
Length of instrument	Small - small number of lines	Moderate – can take time per eye	Small	Small	Moderate	Moderate
Completion time	Within minutes	15-20 minutes	Within minutes	Within minutes	Relative	Relative
Patient mental ability level	Low – use with children	Medium	Low	Low	Medium	Medium
Ease of standardisation	High ease	High	High	High	Moderate	Moderate
Clinician comprehension	Widely understood	Widely understood	Widely understood	Widely understood	Widely understood	Widely understood
Type of instrument	Letter chart	Perimeter	Occluder, target, prisms	Occluder, target	Questionnaire	Reading paragraphs
Cost	Low	High	Low	Low	Relative to licence	Low
Required equipment	Letter chart	Perimeter	Occluder, target, prisms	Occluder, target	Questionnaire	Book

For peer review only - http://bmjopen.bmj.com/site/about/guidelines.xhtml

Type of administration	Clinician-led	Clinician-led	Clinician-led	Clinician-led	Clinician-led Self-administered	Clinician-led
Availability in different settings	Yes	Yes	Yes	Yes	Yes	Yes
Copyright	Per company	Per company	Per company	Per company	Per company	Per company
Patient physical ability level	Minimal	Relative	Minimal	Minimal	Relative	Relative
Regulatory approval	No	No	No	No	No	No
Ease of score calculation	High ease	High ease	High ease	High ease	High ease	High ease

For peer review only

Table 3, Reasons for exclusion of instruments

**Instrument / measurement**	**Exclusions**
Case history	Non-standardised and subjective
Observations	Non-standardised and subjective
Visual acuity (non-logMAR options), e.g.:<br>Snellen charts<br>Fixation and following observation<br>Vanishing optotype charts<br>Grating charts<br>Near acuity charts<br>Kay's pictures<br>Sheridan Gardiner single optotypes<br>Lea symbols	Inconsistencies in measurement properties
Ocular position, e.g.:<br>Krimsky test<br>Prism reflection test<br>Synoptophore<br>Bruckner test<br>Maddox rod	Inconsistencies in measurement properties and inclusion of subjective examiner judgments
Ocular movements, e.g.:<br>Hess/Lees/Harms wall charts	Insufficient widespread access and insufficient assessment of peripheral eye movements
Visual fields:<br>Confrontation<br>Central perimetry	Subjective examiner assessment and/or insufficient assessment of the peripheral field of vision beyond 30 degrees
Reading, e.g.:<br>IReST<br>Wilkins<br>Vocational reading charts	Reading sections set at specific font size<br>Non-adaptable to low vision patients
Activities of daily living, e.g.:<br>ADL (Activities of Daily Living)<br>AMPS (Assessment of Motor and Process Skills)<br><br>FAI (Frenchay Activities Index)<br>FIM (Functional Independence Measure)<br>MET (Multiple Errands Test)<br>RNLI (Reintegration to Normal Living Index)<br><br>SIS (Stroke Impact Scale)<br>LIFE-H (Assessment of Life Habits)<br>ACS (Activity Card Sort)<br>MRS (Modified Rankin Scale)	No one specific test targeting vision-related activities of daily living
Vision-related Quality of life, e.g.:<br>VA LV (Veterans Low Vision Visual Function Questionnaire)<br>AI (Activity Inventory)<br>DLTV (Daily Living Tasks Dependent on Vision)<br><br>SRA-VFP (Self-Reported Assessment of Functional Visual Performance)	Inconsistencies in measurement properties

For peer review only

For peer review only - http://bmjopen.bmj.com/site/about/guidelines.xhtml

Figure 1, Flowchart of COS and COM development process

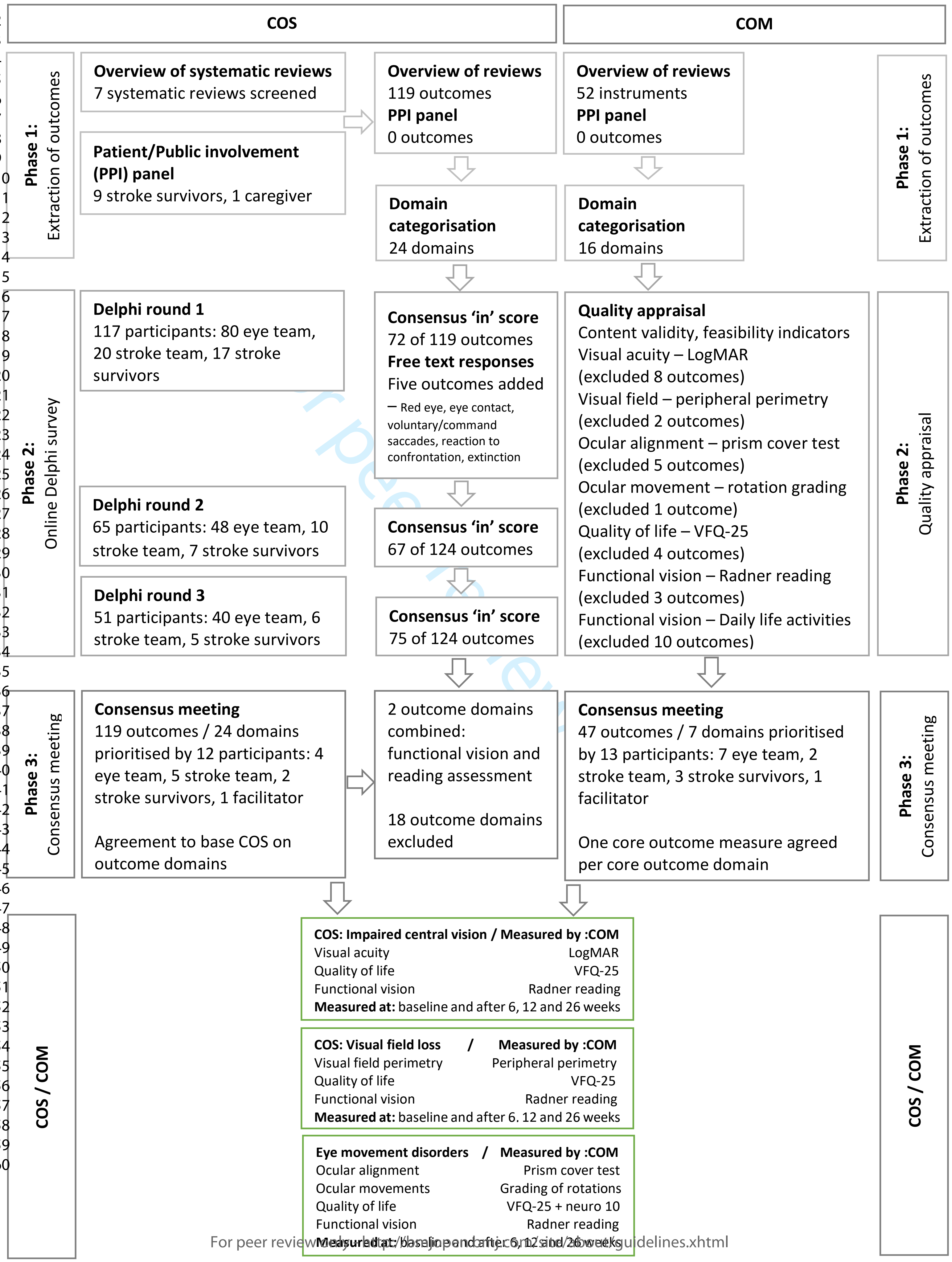

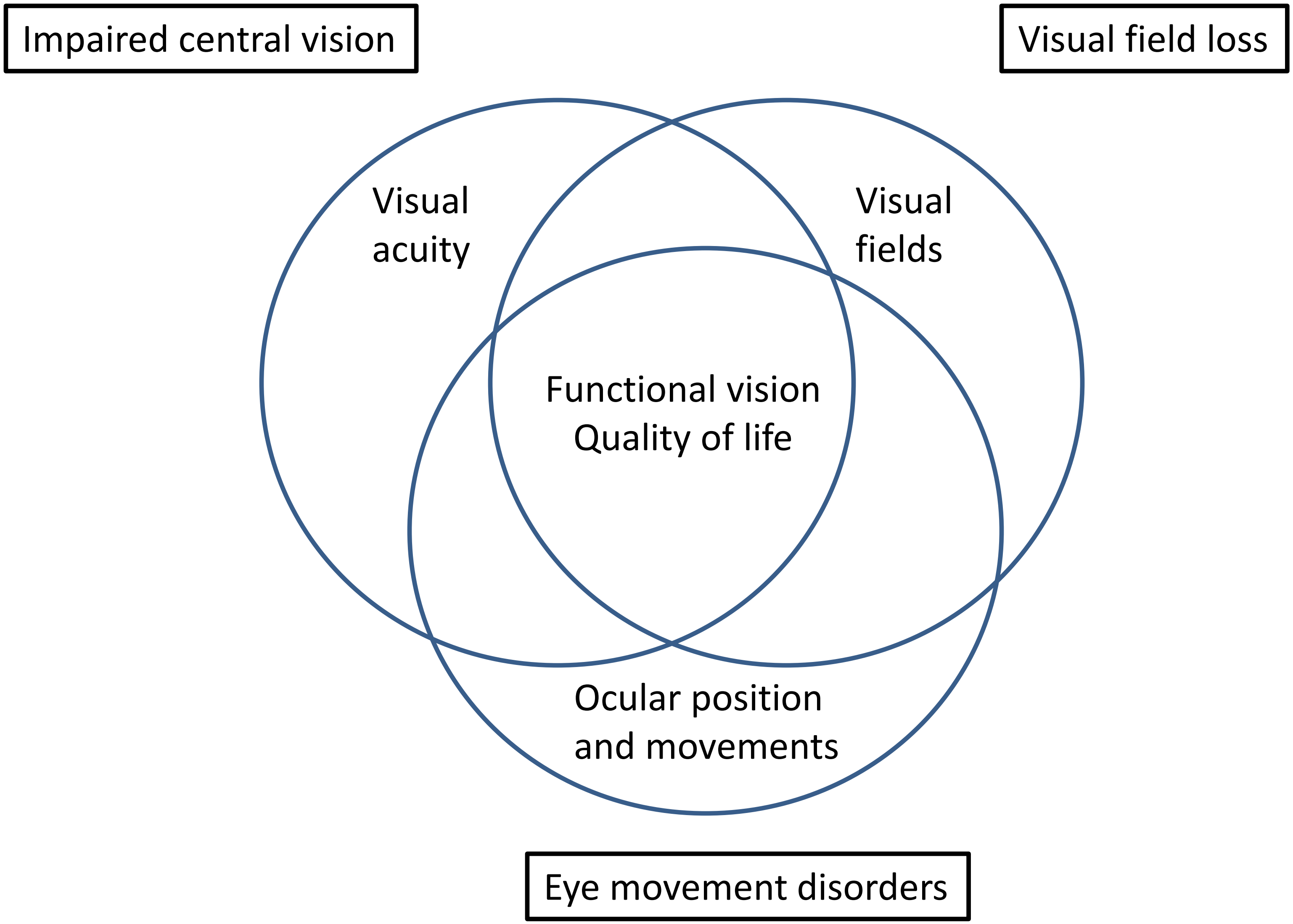
Impaired central vision
Visual field loss
Visual
acuity
Visual
fields
Functional vision
Quality of life
Ocular position
and movements
Eye movement disorders

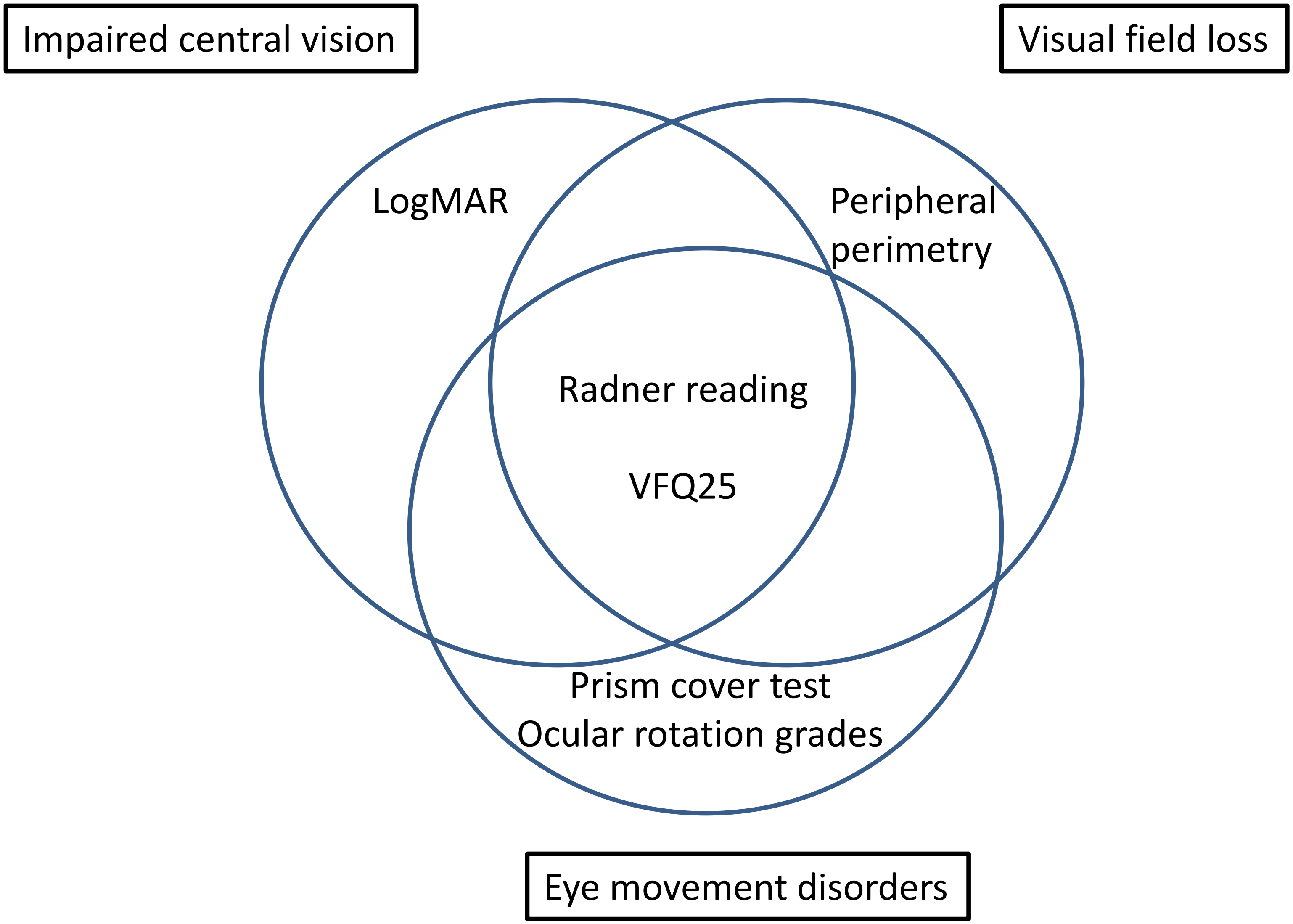
Impaired central vision
Visual field loss
LogMAR
Peripheral perimetry
Radner reading
VFQ25
Prism cover test
Ocular rotation grades
Eye movement disorders

COMET initiative registration

https://www.comet-initiative.org/Studies/Details/1420

Development of core outcome sets for vision screening and assessment in stroke: a Delphi and consensus study

OBJECTIVES: Visual impairment following stroke is common with a reported incidence of visual impairment in 60% of stroke survivors. Screening for visual impairment is neither routine nor standardised. This results in a health inequality where some stroke survivors receive comprehensive vision assessment to identify any existent visual problems while others receive no vision assessment leaving them with unmet needs from undiagnosed visual problems. The aim of this study was to define two core outcome sets (COS), one for vision screening and one for full visual assessment of stroke survivors. DESIGN: A list of potentially relevant visual assessments was created from a review of the literature. The consensus process consisted of an online 3-round Delphi survey followed by a consensus meeting of the key stakeholders. PARTICIPANTS: Stakeholders included orthoptists, occupational therapists, ophthalmologists, stroke survivors and COS users such as researchers, journal editors and guideline developers. SETTING: University. OUTCOME MEASURES: COS. RESULTS: Following the consensus process we recommend the following nine assessments for vision screening: case history, clinical observations of visual signs, visual acuity, eye alignment position, eye movement assessment, visual field assessment, visual neglect assessment, functional vision assessment and reading assessment. We recommend the following 11 assessments for full vision assessment: case history, observations, visual acuity, eye alignment position, eye movement assessment, binocular vision assessment, eye position measurement, visual field assessment, visual neglect assessment, functional vision assessment, reading assessment and quality of life questionnaires. CONCLUSIONS: COS are defined for vision screening and full vision assessment for stroke survivors. There is potential for their use in reducing heterogeneity in routine clinical practice and for improving standardisation and accuracy of vision assessment. Future research is required to evaluate the use of these COS and for further exploration of core outcome measures.

Aim

The aim of this study was to define two core outcome sets (COS), one for vision screening and one for full visual assessment of stroke survivors.

Contributors

Rowe, F. J. Hepworth, L. R. Kirkham, J. J.

Publication

Journal: BMJ Open

Volume: 9

Issue: 9

Pages: e029578 -

Year: 2019

DOI: 10.1136/bmjopen-2019-029578

Further Study Information

Current Stage: Not Applicable

Date:

Funding source(s): This article/paper/report presents independent research funded by the National Institute for Health Research (NIHR: CDF-2012-05-126).

Health Area

Disease Category: Eyes & vision, Neurology

Disease Name: Visual impairment after stroke

Target Population

Age Range: 18 - 100

Sex: Either

Nature of Intervention: Other, Screening

Stakeholders Involved

- Clinical experts
- Consumers (caregivers)
- Consumers (patients)
- Guideline developers
- Journal editors
- Researchers

Study Type

- COS for clinical trials or clinical research
- COS for practice

For peer review only - http://bmjopen.bmj.com/site/about/guidelines.xhtml

Method(s)

- Consensus meeting
- Delphi process
- Literature review

A list of potentially relevant visual assessments was created from a review of the literature. The consensus process consisted of an online 3-round Delphi survey followed by a consensus meeting of the key stakeholders.

For peer review only

BMJ Open

Development of core outcome sets and core outcome measures for central visual impairment, visual field loss and ocular motility disorders due to stroke: a Delphi and consensus study.

Journal:	*BMJ Open*
Manuscript ID	bmjopen-2021-056792.R1
Article Type:	Original research
Date Submitted by the Author:	21-Dec-2021
Complete List of Authors:	Rowe, Fiona; University of Liverpool<br>Hepworth, Lauren; University of Liverpool, Health Services Research<br>Kirkham, Jamie J.; Manchester University, Biostatistics
**Primary Subject Heading**:	Ophthalmology
Secondary Subject Heading:	Health services research
Keywords:	Stroke < NEUROLOGY, STROKE MEDICINE, OPHTHALMOLOGY, Neuro-ophthalmology < OPHTHALMOLOGY

SCHOLARONE™
Manuscripts

I, the Submitting Author has the right to grant and does grant on behalf of all authors of the Work (as defined in the below author licence), an exclusive licence and/or a non-exclusive licence for contributions from authors who are: i) UK Crown employees; ii) where BMJ has agreed a CC-BY licence shall apply, and/or iii) in accordance with the terms applicable for US Federal Government officers or employees acting as part of their official duties; on a worldwide, perpetual, irrevocable, royalty-free basis to BMJ Publishing Group Ltd ("BMJ") its licensees and where the relevant Journal is co-owned by BMJ to the co-owners of the Journal, to publish the Work in this journal and any other BMJ products and to exploit all rights, as set out in our licence.

The Submitting Author accepts and understands that any supply made under these terms is made by BMJ to the Submitting Author unless you are acting as an employee on behalf of your employer or a postgraduate student of an affiliated institution which is paying any applicable article publishing charge ("APC") for Open Access articles. Where the Submitting Author wishes to make the Work available on an Open Access basis (and intends to pay the relevant APC), the terms of reuse of such Open Access shall be governed by a Creative Commons licence – details of these licences and which Creative Commons licence will apply to this Work are set out in our licence referred to above.

Other than as permitted in any relevant BMJ Author's Self Archiving Policies, I confirm this Work has not been accepted for publication elsewhere, is not being considered for publication elsewhere and does not duplicate material already published. I confirm all authors consent to publication of this Work and authorise the granting of this licence.

For peer review only

Development of core outcome sets and core outcome measures for central visual impairment, visual field loss and ocular motility disorders due to stroke: a Delphi and consensus study.

Fiona J Rowe[1], PhD, Lauren R Hepworth[1], PhD, Jamie J Kirkham[2], PhD

1, Institute of Population Health, University of Liverpool, UK

2, Centre for Biostatistics, The University of Manchester, Manchester Academic Health Science Centre, Manchester, UK

Address for correspondence:
Prof Fiona Rowe
Waterhouse Building Block B,
University of Liverpool,
1-3 Brownlow Street,
Liverpool L69 3GL
T: 0151 7944956
E: rowef@liverpool.ac.uk
Twitter: @orthopticbrain

Category: Original research article
Short title: Core outcomes for vision and stroke
Word count: 4542
Number of tables: 3
Number of figures: 3
Supplementary data: 2

For peer review only

Abstract

Objectives: Reporting of research for stroke-related visual impairment is inconsistent. The aim of this study was to define three core outcome sets (COS) and related core outcome measurements (COM) for central visual impairment, visual field loss and ocular motility disorders in stroke research.

Design: The consensus process consisted of an online 3-round Delphi survey followed by a consensus meeting of key stakeholders.

Setting: UK-wide survey.

Participants: Stakeholders included orthoptists, occupational therapists, ophthalmologists, stroke survivors and COS users such as researchers, journal editors and guideline developers.

Outcome measures: For COS development, a list of potentially relevant visual outcomes was created after review of the literature and further grouped into outcome domains. For COM development, a list of potential instruments was created after review of the literature and quality appraised for reliability and validity.

Results: COS - 119 potential outcomes extracted from published literature. Similar assessment outcomes were grouped into 24 outcome domains. Delphi process included 123 participants in round 1, 65 round 2, 51 round 3. Twelve participants attended the consensus meeting with recommended outcome domains for central visual impairment (visual acuity, functional vision, quality of life), visual field loss (visual fields, functional vision, quality of life) and ocular motility disorders (eye alignment, eye movements, functional vision, quality of life). COM - 52 test options extracted from the COS outcomes and grouped into 16 domains. Thirteen participants attended the COM consensus meeting. Recommended instruments for measurement of these outcomes include; LogMAR visual acuity, cover test, cardinal position eye movement assessments, peripheral visual field perimetry, Visual Function Questionnaire-25.

Conclusions: COS and COM are defined for vision research for stroke survivors. Their use has potential to reduce heterogeneity in routine clinical practice and

For peer review only - http://bmjopen.bmj.com/site/about/guidelines.xhtml

improve standardisation and accuracy of vision assessment. Future research is required to evaluate the use of these COS and COM.

Keywords: Core outcome set; Core outcome measures; Vision; Stroke; Delphi; Consensus; Central vision; Visual field loss; Ocular motility

Strengths and limitations of this study

- We report a new core outcome set (COS) and core outcome measures (COM) for stroke-vision research.
- The COS and COM have a break-down for category of visual impairment; specifically, central vision impairment, visual field loss and ocular motility disorders.
- Composite outcome domains were ranked by Delphi and consensus opinion from multi-disciplinary experts and stroke survivors.
- This was not an international study; participants were from UK and Ireland.
- We experienced attrition bias across the Delphi rounds.

For peer review only - http://bmjopen.bmj.com/site/about/guidelines.xhtml

Introduction

Visual impairment is common as a consequence to stroke with a reported incidence of 60% in stroke survivors [1]. Types of visual impairment generally fall into one of four categories including impairments of central vision, eye movements, visual fields and visual perception [2]. The impact of visual impairment on stroke survivors is wide reaching. Visual impairment can increase risk of collisions and falls, impede activities of daily life such as reading and writing, prevent return to work, pre-stroke activities and hobbies, and driving [3-6]. Such impacts result in loss of independence, increase likelihood of social isolation and results in mood change, anxiety and depression [5,6]. There are several approaches to the management of these visual impairments including visual scanning therapy, prisms, occlusion, spectacles, drugs, surgery, botulinum toxin, exercises, or a combination of two or more of the above [7-9].

A range of systematic reviews in the field of stroke-vision research have identified that there is considerable variation in the outcomes being measured and reported in primary research studies, which impacts the ability to compare and synthesise outcome results across studies. Moreover, it was noted that there is a paucity of outcome data available on important patient outcomes such as quality of life and functional visual assessment [7-11].

To mitigate these issues and to increase the relevance of research, a core outcome set (COS) and core outcome measurements (COM) can be developed which represents an agreed standardised set of outcomes and instruments that should be used and reported in all studies for a specific area of health or healthcare [12,13]. A search of the COMET (Core Outcome Measures in Effectiveness Trials) database revealed that there are several studies that have investigated important outcomes for eyes and vision disease; examples include cataracts and glaucoma but none has specifically looked at stroke [14].

The aim of this study was to achieve consensus on the content of vision research outcomes for stroke survivors. In this study we report the results of a Delphi process and consensus meetings in the development of three COS and COM for stroke-

For peer review only - http://bmjopen.bmj.com/site/about/guidelines.xhtml

vision research for the categories of impaired central vision, visual field loss and ocular motility disorders.

Methods

Ethical approval

This study had institutional ethical approval (Ref-1415-040) and was undertaken in accordance with the Tenets of Helsinki. Informed consent was obtained for the Delphi survey from all participants in the consensus meeting.

Development of the COS involved three phases: (1) the generation of a comprehensive list of outcomes; (2) a Delphi survey involving three rounds to gain consensus as to which outcomes are most important; and (3) patient and professional consensus meetings to agree on a final COS.

Development of the COM involved three phases: (1) the generation of a comprehensive list of outcome measurement instruments specific to the outcomes in each COS, (2) quality appraisal of the outcome measurement instruments and (3) a consensus process on recommendations for the selection of outcome measurement instruments.

A protocol for the development of this COS project was written by the steering committee, registered in the COMET initiative website (http://www.comet-initiative.org/studies/details/275?result=true) and available as open access (http://pcwww.liv.ac.uk/~rowef/index_files/Page356.html - supplementary file). When developing this COS, we followed the minimum set of development standards set out by COS-STAD (Core Outcome Set-STAndards for Development) Statement and COSMIN (Consensus-based Standards for the selection of health Measurement Instruments), and report the results against the COS-STAR (Core Outcome Set–STAndards for Reporting) guideline [13,15,16].

The full methods for the COS have been fully outlined previously [17] and detail the steering group, patient and public involvement, and stakeholder groups.

Patient and public involvement

For peer review only - http://bmjopen.bmj.com/site/about/guidelines.xhtml

This study was supported by a patient advisory group (the VISable patient and public involvement panel) which provided input to this research study. The VISable panel met on a regular basis during the conduct of the study. Patients contributed to the design of the study and were involved at all stages of the survey and consensus meeting.

Core outcome set

Phase 1 – Outcome identification

A literature review was conducted to develop a preliminary list of outcomes for the Delphi survey. We undertook an overview of seven systematic reviews of studies/trials reporting vision screening, assessment and treatment of post-stroke visual impairment [2,7-11,18-20] and extracted a list of included outcomes. The list was then circulated to the VISable patient and public involvement panel [21] for approval and checking of writing for lay terms as the basis of the online survey development. VISable (a panel of ten patients/carers) were asked to consider whether any further outcomes should be added, particularly those deemed relevant to patient-reported outcomes. The panel advised that appropriate patient-important outcomes were included already in the list and did not add any further outcomes.

Phase 2 – Delphi survey

We undertook a prospective consensus study using a Delphi process. SurveyMonkey (SurveyMonkey Inc. Palo Alto, California, USA 2015) was used as the online platform to administer the Delphi process. The survey was piloted for checks of ambiguity and appropriate use of lay language by the steering group and then released live. The full survey was circulated through professional, clinical and research networks in the UK and Ireland to reach stakeholder groups of eye care teams (orthoptists, ophthalmologists), stroke teams (physicians, nurses, therapists) and patients (stroke survivors).

The Delphi survey consisted of three rounds. Rounds remained open for 10 weeks, with regular two-week reminders sent to those that had partially completed or not completed the survey in order to maximise response rates. All terms had explanatory

For peer review only - http://bmjopen.bmj.com/site/about/guidelines.xhtml

notes to aid interpretation. All participants were asked to score each outcome in terms of importance and asked to identify any additional outcomes of importance that did not appear in the list of assessments. Additional outcomes added in round 1 were reviewed by the steering group to consider their relevance to the survey and to identify and remove duplicates.

Scoring Method: In each round, participants were asked to score the importance of each outcome listed on a nine-point scale (1-3: not important; 4-6: important but not critical; 7-9: critical) as well as an 'unable to score' option. The scale was devised by the GRADE working group [22] to score the quality of evidence for outcomes in systematic reviews and has been adopted in other core outcome set development work research using Delphi methods.

Methods of analysis: For each round of Delphi, the results for each outcome were summarised, which included providing the number of participants responding to the survey, as well as the mean, standard deviation and the percentage of participants scoring the outcome at each possible level from 1-9.

In round 2, all data from round 1 was analysed and compiled by stakeholder groups;1) stroke survivors/carers, 2) stroke team professionals and 3) eye team professionals, to allow different perspectives to be considered prior to re-rating [12]. The summarised results for each of the three stakeholder groups were provided to all participants in the form of histogram plots. Each participant was also shown their personal original score for each outcome. Participants had the opportunity to re-score based on the summary scores from the three stakeholder groups versus their previous personal scores. All participants were also asked to score on additional outcomes that were identified and added in round 1.

In round 3, the results from round 2 were analysed. Following analysis, the results from each stakeholder group were reviewed by the steering group and judged to be similar in terms of mean responses and percentage spread across the responses of 1 to 9. Thus, they were presented as one compilation of all responses. Each participant was shown their personal score from round 2 for each outcome along with the summary scores from all other participants, and asked to score each outcome again in terms of importance.

Consensus was defined *'a priori'* however, this information was not provided on the Delphi survey. Participants were aware of these cut off values at the time of

For peer review only - http://bmjopen.bmj.com/site/about/guidelines.xhtml

attending the consensus meeting and these values were applied overall to responses: 'Consensus in' (i.e. consensus that the outcome should be included in the core set) was defined as greater than 70% of participants scoring as 7-9 and less than 15% of participants scoring 1-3. 'Consensus out' (i.e. consensus that the outcome should not be included in the core set) was defined as greater than 70% of participants scoring as 1-3 and less than 15% of participants scoring as 7-9. All other combinations were seen as equivocal. The outcomes that were designated as 'consensus in' or seen as 'equivocal' were taken forward and discussed in more detail at the consensus meeting for inclusion into the final core outcome sets. Participants were reminded of all outcomes not reaching consensus as part of the Delphi process.

Phase 3 – Consensus meeting

All participants from the Delphi survey were invited to contribute to the consensus meeting. Those expressing interest were invited to attend a face to face consensus meeting. All round 3 survey completers were emailed an invitation. Our intention was to ensure all stakeholders were reasonably represented. The format of the meeting included a short study overview which outlined the categories of visual impairment due to stroke and outcomes identified from the outcome identification phase, a presentation containing a summary of the results and number of outcomes reaching consensus from the survey. Each outcome was to be considered in turn, in order of their presentation in the Delphi survey, to ratify these results. Each remaining outcome was then be considered in turn with full discussion. Similar to the survey, each outcome was considered as reaching consensus with 70% of participants voting in favour of its inclusion.

Each participant voted on every outcome being asked to vote 'Yes' (this outcome should be included in the COS), 'No' (this outcome should not be included), or 'Unsure' using voting slips. Results were collated after voting for all outcomes was completed and results then presented to the group. Outcomes were retained or dropped when consensus is reached. Discussion and further rounds of voting, restricting the options to 'Yes' or 'No' was undertaken until consensus was reached on all outcomes. All outcomes retained were to be included in the final COS.

For peer review only

Core outcome measures

Phase 1 – Outcome identification

From the same literature review as the COS, a comprehensive list of outcome measurement instruments was generated, with circulation of the list to the VISable panel for review.

Phase 2 – Quality appraisal

We undertook a quality appraisal of the available outcome measurements identified from the literature review. For each outcome measurement instrument, we conducted a further review of the medical literature to seek evidence of the use of the instrument in a stroke population. We evaluated the validity of the instrument and feasibility for its use with stroke survivors as per COSMIN guidance [13]. Measurement properties included content validity, internal structure, reliability, measurement error, hypotheses testing, cross-culture validity, criterion validity and responsiveness. Feasibility aspects included comprehensibility, interpretability, ease and type of administration, length of the instrument, completion time, patient mental and physical ability level, ease of standardisation and scoring, type of instrument, cost, equipment required, availability in different settings, copyright, and regulatory approval requirements. Quality of evidence was graded from low to high.

Phase 3 – Consensus meeting

A subsequent (and separate to COS meeting) consensus meeting was planned for discussion of the COM development. Representatives from the COS Delphi survey were invited to a face to face consensus meeting; all round 3 survey completers were emailed an invitation. The intention was to ensure all stakeholders were reasonably represented. The same process as outlined above for COS development was followed for introduction to the process, definition of consensus, voting, and compilation of results. The plan was to seek consensus agreement on the inclusion of one instrument for each category of stroke-visual impairment research; specifically, categories of impaired central vision, ocular motility disorders and visual field loss. Consensus agreement was only sought for the agreed COS outcomes. Further, consensus agreement was sought on the timing of measurement.

For peer review only - http://bmjopen.bmj.com/site/about/guidelines.xhtml

Results

Figure 1 outlines a flowchart of the results for number of participants and number of outcomes in the development and agreement of the COS and COM.

Core outcome set process

Phase 1 - Outcome identification

We extracted 119 outcomes, many of which were variations on assessments for specific visual functions. For the purposes of clarity during COS development, similar assessments were also combined into outcome domains. This process produced a list of 24 domains (Table 1).

Phase 2 – Delphi survey

In total 123 participants registered for round 1 of the Delphi survey. Six registered but did not answer any survey questions. The remaining participants comprised of 79 orthoptists, 20 occupational therapists (stroke team stakeholder group), 17 stroke survivors (stroke survivor/carer stakeholder group) and 1 ophthalmologist. The orthoptists and ophthalmologist formed the eye team stakeholder group. There were 20 males and 97 females with ages ranging from 18-84 years. In round 2, 65 participants completed the survey – an attrition of 44.5% from round 1. These participants comprised 47 orthoptists, 10 occupational therapists, 7 stroke survivors and 1 ophthalmologist. In round 3, 51 participants completed the survey – an attrition of 56.4% from round 1 (21.5% from round 2). These participants comprised of 39 orthoptists, 6 occupational therapists, 5 stroke survivors and 1 ophthalmologist.

Following completion of the survey of 119 outcomes in round 1, five additional outcomes were put forward by the participants for inclusion in round 2: red eye, eye contact, reaction to confrontation, visual extinction and voluntary/command saccades. Consensus 'in' scores were achieved for 72 outcomes. No outcomes scored less than 3. After round 2 of 124 outcomes, no new outcomes were introduced. Consensus 'in' scores were achieved for 67 outcomes. No outcomes scored less than 3. Following round 3 of 124 outcomes, consensus 'in' scores were achieved for 75 outcomes. The Delphi survey reached the following consensus:

For peer review only - http://bmjopen.bmj.com/site/about/guidelines.xhtml

- Full consensus for inclusion was obtained for case history asking patients/carers open and specific questions plus checks of previous ocular history and glasses wear. Full consensus for observations, tests of visual acuity, eye alignment, eye movements, binocular vision, alignment measurement, visual fields, visual inattention, reading, lids and pupils, visual perception and functional visual assessment.
- No consensus was reached for eye contact during conversations, use of questionnaires, contrast sensitivity, colour vision, fundus checks and use of retinal photography/optical coherence tomography.

Phase 3 – Consensus meeting

The consensus meeting was a 1-day event held in Liverpool, UK with 12 participants comprising 5 occupational therapists (2 with research roles), 3 orthoptists (1 with a research role), 2 patients, 1 Cochrane editor and 1 facilitator. The facilitator did not take part in the voting. The objective of the meeting was to discuss and vote on the Delphi outcomes – the results of the Delphi survey had been provided to participants prior to and during the meeting.

During the consensus meeting, final consensus for research vision assessment was split according to category of visual impairment, (impaired central vision, visual field loss and ocular motility disorders). Participants decided to organise the COS on outcome domains rather than specific outcomes on the basis that many outcomes were similar and were better represented as a grouped outcome domain (see grouping of outcomes in Table 1). Two outcome domains were combined because of overlap in outcomes: functional vision and reading assessments.

No consensus could be reached for visual inattention or for visual perceptual disorders. Visual perceptual disorders were considered too heterogenous with so many different types of visual perception issue occurring. It was therefore not possible to determine a core set of assessments. Visual inattention was considered too broad and variable a condition with a discussion amongst consensus meeting participants about context and timing of assessment for stroke survivors with visual attention. Agreement was therefore obtained only for categories of impaired central vision, visual field loss and eye movement disorders (Figure 2). Impaired central vision consisted of visual acuity, functional vision and quality of life. Visual field loss

For peer review only - http://bmjopen.bmj.com/site/about/guidelines.xhtml

For peer review only

consisted of visual field perimetry, functional vision and quality of life. Eye movement disorders consisted of ocular alignment, ocular movement, functional vision and quality of life.

Core outcome measures process

Phase 1 - Outcome identification

From 119 outcomes identified for the COS, we extracted 52 test options. For the purposes of clarity, similar tests were combined into outcome domains. This process produced a list of 16 domains (Table 1).

Phase 2 – Quality appraisal

Quality assessment was conducted for the available outcome measurement instruments considering their measurement properties and feasibility aspects. Table 2 outlines the appraisal summary for selected instruments meeting quality indicators specific to the outcomes selected for the three COS of central vision impairment, visual field loss and ocular motility disorders; Logarithm of the Minimal Angle of Resolution (LogMAR) visual acuity, peripheral perimetry, prism cover test, ocular rotation grading, vision-related quality of life and reading.

Phase 3 – Consensus meeting

The consensus meeting was held as an online event with 13 participants comprising 2 occupational therapists, 7 orthoptists (2 with a research role), 3 patients and 1 facilitator. The facilitator did not take part in the voting. The objective of the meeting was to discuss and vote on the COM – the results of a comprehensive list of outcome measurements and their quality appraisal had been provided to participants prior to and during the meeting. Each participant was invited to make queries for each instrument and seek clarification of any part of the quality appraisal. Agreement was requested from each participant on inclusion of each selected instrument.

During the consensus meeting, final consensus for research vision assessment was split according to type of measurement (Figure 3). Overall excluded instruments and reasons for exclusion are outlined in table 3. Recommendation for inclusion was obtained for;

For peer review only - http://bmjopen.bmj.com/site/about/guidelines.xhtml

- LogMAR test (impaired central vision). This test may require the use of a matching card because of communication or cognitive issues.
- Peripheral perimetry (visual field loss). Specifically, peripheral visual field testing was recommended over central visual field testing to acknowledge the need to detect presence of peripheral visual field loss outside the central 30 degrees and detection of change occurring in the peripheral visual field over time. Kinetic perimetry was recommended with the additional of static points to check the integrity of the visual field within the peripheral boundary. Where static-only programmes were available, a recommendation was made to use the Esterman programme which is most commonly available regardless of equipment type. A recommendation was made to use the binocular Esterman programme as the minimum core assessment but with the addition of monocular Esterman fields for each eye where possible dependent on patient ability.
- Prism cover test and ocular rotation grading (ocular movement disorders). Ocular alignment determined by the cover test and measured by prism cover test. Ocular rotation gradings specifically in nine positions of gaze and assessed using saccadic and smooth pursuit movements alongside vergence (convergence and divergence). Recommendation to incorporate vestibulo-ocular reflex movements and/or optokinetic nystagmus where relevant (e.g. if smooth pursuit movements cannot be fully examined).
- VFQ-25 questionnaire (vision-related quality of life) [23]. Recommendation to add neuro-10 supplement when used for ocular motility COS but not appropriate for use in impaired central vision or visual field loss COS.
- Radner test (functional vision: reading impairment) [24]. Recommendation to capture additional impact to reading by non-visual issues such as presence of aphasia.

No consensus could be reached for functional vision assessment incorporating activities of daily living. Functional visual assessment was considered too heterogenous for types of assessment in relation to visual function versus general function and therefore it was not possible to determine one, or even a small number, of measurement tools. Consensus was reached that the aspect of functional vision with respect to activities of daily living warrants further research and should consider

For peer review only - http://bmjopen.bmj.com/site/about/guidelines.xhtml

the issues identified during the Delphi process and focus groups, including; observed navigation, eye scanning, walking observations, activities of daily living, self-care, body placement, spatial awareness, mobility observations, writing, hand-eye coordination, visual memory and cognition and visual perception.

The final stage of the consensus meeting was to consider the time points at which the COM should be made as a minimum. Consensus was achieved for a recommended measurement timescale of baseline and follow-up periods of 4 weeks, 12 weeks and 26 weeks, for all three COS. There was a recommendation that consideration should be given to recruitment in acute versus chronic stages of stroke given that rapid changes may occur in visual function within the first month of stroke onset.

Discussion

A core outcome set (COS) is an agreed minimum set of outcome measures that should be reported. By reporting a minimum set of measures, this reduces the heterogeneity of outcomes across studies, which in turn supports future evidence synthesis [12,15,16]. Once a COS is defined, it is important to achieve consensus on how the outcomes should be measured [13]. Core outcome measurements (COM) define how to measure core outcomes and are the instruments selected to measure each core outcome [13].

We have previously used Delphi and consensus process methods to develop test batteries for general clinical vision screening and assessment for post-stroke visual impairment [17]. The purpose of this present study was to agree on a COS and COM specific to use in stroke-vision research, distinct from routine clinical screening and assessment practice. After round 3 and the consensus meeting, stakeholders agreed with the proposed COS for vision research for three categories of visual impairment, consisting of three outcome domains for impaired central vision, three for visual field loss and four for eye movement disorders. Core amongst all categories were the domains of functional vision and quality of life. It is important to acknowledge these COS do not exclude further visual assessments which should be added as appropriate and relevant for the individual research study. Indeed, it is essential to

For peer review only - http://bmjopen.bmj.com/site/about/guidelines.xhtml

ensure that the multiple visual morbidities that arise due to stroke, are also properly recognised, sought and reported, despite not being selected as core elements.

Generally, one instrument is selected for each COM [13]. For assessment of impaired central vision, the selected instrument to test visual acuity was a logMAR chart, for visual field loss, peripheral perimetry was selected and for eye movement disorders, the selected instrument was the prism cover test when measuring ocular alignment, and grading of ocular rotations for measuring eye movement. For assessment of vision-related quality of life, the VFQ-25 questionnaire was selected and for functional vision assessment specific to reading, the Radner reading test was selected. It was not possible to reach consensus for measurement of other aspects of functional vision. There was unanimous agreement that further research is required to evaluate measurements of activities of daily living in relation to vision versus general function with specific aspects to be considered such as observed navigation and mobility, spatial awareness, self-care and writing. Quality appraisal for COM considered documentation of the use of the instrument in the target population, content validity, quality of evidence, and feasibility of use of the instrument [25-50]. Timing of these measurements were recommended at minimum time points of baseline and follow-up at 4, 12 and 26 weeks.

There are a number of strengths for this study. A COS has been produced for stroke-vision research with a break-down for category of visual impairment; specifically, central vision impairment, visual field loss and ocular motility disorders. This addresses a gap in evidence-based practice for post-stroke visual impairment. The COS is composed of outcome domains which were ranked by Delphi and consensus opinion from multi-disciplinary experts and stroke survivors. Furthermore, they are based on existent outcomes for which there are assessment instruments (i.e. COM) that are easily accessible for implementation, and accepted as validated clinical measures. Although these COS and COM were developed for stroke, there is potential for their use with other types of acquired brain injury causing visual impairment.

There are limitations to this study. Despite circulation of the survey across a wide range of networks, not all members of eye care and stroke teams took part despite invitation, e.g. lack of optometry, neuropsychology input, ophthalmology / neuro-

For peer review only - http://bmjopen.bmj.com/site/about/guidelines.xhtml

ophthalmology. Participants were from UK and Ireland and largely represented views and practices within the National Health Service (NHS). Thus, assessments are those used clinically in these countries. However, we are aware that the assessment outcome domains selected for the COS and instruments selected for the COM are those that are widely used internationally in clinical and research settings as evidenced from our initial literature review [2,8,10]. Indeed, the quality appraisal of outcome measurement instruments involved many studies undertaken in countries other than the UK [25-50]. We experienced attrition bias across the Delphi rounds. The attrition rate at round 2 was 44.5% and, by round 3, was 56.4% from the initial sample. High attrition rates however are common using Delphi methods and our rates can be considered within an acceptable range based on those reported for other COS developments [51]. Nevertheless a larger sample would have been preferable. Researcher bias is also a potential limitation. We aimed to limit this by providing a summary of results across all rounds of the Delphi survey and with final decisions left to the consensus meeting with experts. There can be risks from using Delphi methods in which participants can have very disparate views of each outcome. However, we sought a wide variety of participants across a number of stakeholder groups to achieve greater consistency in responses and balance potential outlier responses. Further, this core outcome development process included a final stage of consensus meetings such that decisions were not purely made from the Delphi responses. Although we initially included outcomes for visual neglect/inattention and visual perception, it was not possible to reach consensus for a COS for these outcomes through the Delphi survey or consensus process. A detailed discussion took place during the consensus process that highlighted the specific issues for these outcomes and, in particular, the issue of context of assessment (where, when) and type (personal/peri-personal/extra-personal) of visual neglect, and significance of heterogeneity for forms of other visual perceptual disorders. Further it was not possible to reach consensus for a COM for functional vision specific to activities of daily living. Discussion during the consensus meeting highlighted that no specific instrument/tool for activities of daily living focussed on functional vision and that this represented a gap in evidence requiring further research. Whilst visual inattention, visual perception disorders and functional visual assessment were not included in the COS and COM developed in this study, these are topics that warrant further research to target core outcomes and measurements.

For peer review only - http://bmjopen.bmj.com/site/about/guidelines.xhtml

These disorders can be debilitating consequences of stroke affecting the visual regions of the brain and we advocate for further research on development of COS and COM as essential for these disorders.

Conclusions

This study reports the use of Delphi and consensus methods in the development of a core outcome set and core outcome measurements for stroke-vision research. Impaired central vision and visual field loss each comprised three outcome domains and eye movement disorders comprised four outcome domains. Each COS outcome domain has one recommended outcome measurement instrument except for functional vision relating to activities of daily living for which no consensus could be reached. These core outcome sets and core outcome measurements will facilitate standardisation of stroke-vision assessment in order to reduce heterogeneity in assessment in future research. Further research is now required to evaluate the use of these core outcome sets and outcome measures.

Acknowledgements

We thank all participants involved in the Delphi survey and consensus meetings. We also thank our clinical and patient colleagues who contributed to the study steering group.

Declarations of interest

Funding: This article/paper/report presents independent research funded by the National Institute for Health Research (NIHR: CDF-2012-05-126). The views expressed are those of the authors and not necessarily those of the NHS, the NIHR or the Department of Health.

Conflicts of interest: All authors declare: no financial relationships with any organisations that might have an interest in the submitted work in the previous three years; no other relationships or activities that could appear to have influenced the submitted work.

For peer review only - http://bmjopen.bmj.com/site/about/guidelines.xhtml

Data availability: The Delphi dataset is available from the lead author on reasonable request.

Ethics approval: This study had institutional ethical approval (Institute of Population Health Ethics Committee; Ref-1415-040) and was undertaken in accordance with the Tenets of Helsinki. Informed consent was obtained for the Delphi survey and from all participants in the consensus meeting.

Author contributions: FR provided oversight for the study and led the writing of the paper. FR, LH and JK contributed to data collection, reviewing the draft paper and approving the final version.

References

1. Rowe FJ, Hepworth LR, Howard C, Hanna KL, Cheyne CP, Currie J (2019) High incidence and prevalence of visual problems after acute stroke: An epidemiology study with implications for service delivery. PLoS ONE 14(3): e0213035. https://doi.org/10.1371/journal.pone.0213035
2. Hepworth LR, Rowe FJ, Walker MF, Rockliffe J, Noonan C, Howard C, Currie J. Post-stroke Visual Impairment: A Systematic Literature Review of Types and Recovery of Visual Conditions. Ophthalmology Research: An International Journal. 2015; 5(1). ISSN: 2321-7227
3. Hepworth L, Rowe FJ. Visual impairment following stroke – the impact on quality of life: a systematic review. Ophthalmology Research: an international journal. 2016; 5(2): 1-15
4. Jones SA, Shinton RA. Improving outcome in stroke patients with visual problems. Age Ageing. 2006; 35:560-5.
5. Tsai SY, Cheng CY, Hsu WM, Su TP, Liu JH, Chou P. Association between visual impairment and depression in the elderly. Journal of the Formosan Medical Association. 2003;102:86-90.
6. Chia E-M, Wang JJ, Rochtchina E, Smith W, Cumming RR, Mitchell P. Impact of bilateral visual impairment on health-related quality of life: The Blue Mountains eye study. Investigative Ophthalmology & Visual Science. 2004;45(1):71-76.
7. Pollock A, Hazelton C, Henderson CA, Angilley J, Dhillon B, Langhorne P, Livingstone K, Munro FA, Orr H, Rowe FJ, Shahani U. Interventions for visual

For peer review only - http://bmjopen.bmj.com/site/about/guidelines.xhtml

field defects in patients with stroke. Cochrane Database of Systematic Reviews 2011, Issue 10. Art. No.: CD008388. DOI: 10.1002/14651858.CD008388.pub2.

8. Hanna KL, Hepworth LR, Rowe FJ. The treatment methods for post-stroke visual impairment: a systematic review. Brain and Behaviour. 2017; 7(5): e00682. DOI: 10.1002/brb3.682
9. Bowen A, Hazelton C, Pollock A, Lincoln NB. Cognitive rehabilitation for spatial neglect following stroke. Cochrane Database of Systematic Reviews 2013, Issue 7. Art. No.: CD003586. DOI: 10.1002/14651858.CD003586.pub3
10. Hanna KL, Hepworth R, Rowe FJ. Screening methods for post stroke visual impairment; a systematic review. Disability and Rehabilitation. 2016: DOI: 10.1080/09638288.2016.1231846
11. Hepworth L, Rowe FJ, Harper R, Jarvis K, Shipman T, Rodgers H. Patient reported outcome measures for visual impairment after stroke: a systematic review. Health and Quality of Life Outcomes. 2015; DOI: 10.1186/s12955-015-0338-x
12. Williamson PR, Altman DG, Bagley H, Barnes KL, Blazeby JM, Brookes ST, Clarke M, Gargon E, Gorst S, Harman N *et al*: The COMET Handbook: version 1.0. Trials 2017, 18(3):280.
13. Prinsen CAC, Vohra S, Rose MR, Boers M, Tugwell P, Clarke M, Williamson PR, Terwee CB. How to select outcome measurement instruments for outcomes included in a 'Core Outcome Set' – a practical guideline. Trials. 2016; 17: 449. DOI: 10.1186/s13063-016-1555-2
14. COMET-initiative. Core Outcome Measures in Effectiveness Trials. http://www.comet-initiative.org/ (last accessed 4th September 2020)
15. Kirkham JJ, Davis K, Altman DG, Blazeby JM, Clarke M, Tunis S, et al. (2017) Core Outcome Set-STAndards for Development: The COS-STAD recommendations. PLoS Med 14(11): e1002447. https://doi.org/10.1371/journal.pmed.1002447
16. Kirkham JJ, Gorst S, Altman DG, Blazeby JM, Clarke M, Devane D, et al. (2016) Core Outcome Set–STAndards for Reporting: The COS-STAR Statement. PLoS Med 13(10): e1002148. https://doi.org/10.1371/journal.pmed.1002148

For peer review only - http://bmjopen.bmj.com/site/about/guidelines.xhtml

17. Rowe FJ, Hepworth LR, Kirkham JJ. Development of core outcome sets for vision screening and assessment in stroke: a Delphi and consensus study. *BMJ Open* 2019;9:e029578. doi: 10.1136/bmjopen-2019-029578
18. Pollock A, Hazelton C, Henderson CA, Angilley J, Dhillon B, Langhorne P, Livingstone K, Munro FA, Orr H, Rowe FJ, Shahani U. Interventions for age-related visual problems in patients with stroke. Cochrane Database of Systematic Reviews 2012, Issue 3. Art. No.: CD008390. DOI: 10.1002/14651858.CD008390.pub2
19. Pollock A, Hazelton C, Henderson CA, Angilley J, Dhillon B, Langhorne P, Livingstone K, Munro FA, Orr H, Rowe FJ, Shahani U. Interventions for disorders of eye movement in patients with stroke. Cochrane Database of Systematic Reviews 2011, Issue 10. Art. No.: CD008389. DOI: 10.1002/14651858.CD008389.pub2.
20. Hanna KL, Rowe FJ. Clinical versus evidence-based rehabilitation options for post-stroke visual impairment. Neuro-Ophthalmology. 2017; epub. http://dx.doi.org/10.1080/01658107.2017.1337159
21. The Vision and Stroke patient and public group (VISable). www.vision-research.co.uk (last accessed 4th September 2020)
22. www.gradeworkinggroup.org (last accessed 4th September 2020)
23. Raphael BA, Galetta KM, Jacobs DA, Markowitz CE, Liu GT, Nano-Schiavi ML, et al. Validation and test characteristics of a 10-item neuro-ophthalmic supplement to the NEI-VFQ-25. American Journal of Ophthalmology. 2006;142(6):1026–35
24. Radner W. Reading charts in ophthalmology. Graefes Archives of Clinical and Experimental Ophthalmology. 2017; 94: 714-724
25. Rowe FJ, VIS Group. Vision in Stroke cohort: profile overview of visual impairment. Brain and Behaviour. 2017*;e00771.* https://doi.org/10.1002/brb3.771
26. Rowe FJ, Conroy EJ, Bedson E, Cwiklinski E, Drummond A, García- Fiñana M, Howard C, Pollock A, Shipman T, Dodridge C, MacIntosh C, Johnson S, Noonan C, Barton G, Sackley C. A pilot randomised controlled trial comparing effectiveness of prism glasses, visual search training and standard care in hemianopia. Acta Neurologica Scandinavica. 2016; epub: DOI: 10.1111/ane.12725

For peer review only

For peer review only - http://bmjopen.bmj.com/site/about/guidelines.xhtml

27. Manny RE, Hussein M, Gwiazda J, Marsh-Tootle W, COMET study group. Repeatability of ETDRS visual acuity in children. Investigative Ophthalmology and Visual Science. 2003; 44: 3294-300
28. Stewart CE. LogMAR visual acuity measurements: limits of normality. British and Irish Orthoptic Journal. 2006; 3: 9-13
29. Negiloni K, Mazumdar D, Neog A, et al. Construction and validation of logMAR visual acuity charts in seven Indian languages. *Indian J Ophthalmol*. 2018;66(5):641-646. doi:10.4103/ijo.IJO_1165_17
30. Lam AK, Tong C, Tse J, Yu M. Repeatability of near visual acuity measurement at high and low contrast. *Clin Exp Optom*. 2008;91(5):447-452. doi:10.1111/j.1444-0938.2007.00235.x
31. Ferris FL 3rd, Bailey I. Standardizing the Measurement of Visual Acuity for Clinical Research Studies: Guidelines From the Eye Care Technology Forum. Ophthalmology. 1996 Jan;103(1):181-2.
32. Lempert P. Standardized Measurements of Visual Acuity for Clinical Research. Ophthalmology. 1996 Jul;103(7):994-5.
33. Ricci F, Cedrone C, Cerulli L. Standardized measurement of visual acuity. Ophthalmic Epidemiol. 1998 Mar;5(1):41-53.
34. Hull S, Tailor V, Balduzzi S, Rahi J, Schmucker C, Virgili G, Dahlmann-Noor A. Tests for detecting strabismus in children aged 1 to 6 years in the community. Cochrane Database of Systematic Reviews 2017, Issue 11. Art. No.: CD011221. DOI: 10.1002/14651858.CD011221.pub2.
35. Rowe FJ, Noonan CP, Manuel M. Comparison of Octopus Semi-Automated Kinetic Perimetry and Humphrey Peripheral Static Perimetry in Neuro-Ophthalmic Cases. ISRN Ophthalmology, vol. 2013, Article ID 753202. doi:10.1155/2013/753202
36. Rowe FJ, Rowlands A. Comparison of diagnostic accuracy between Octopus 900 and Goldmann kinetic visual fields. BioMed Research International. Volume 2014, Article ID 214829, doi: 10.1155/2014/214829
37. Ramirez AM, Chaya CJ, Gordon LK, Giaconi JA. (2008) A comparison of semiautomated versus manual Goldmann kinetic perimetry in patients with visually significant glaucoma. Journal of Glaucoma. 17: 111-117

For peer review only - http://bmjopen.bmj.com/site/about/guidelines.xhtml

38. Johnson CA. Standardizing the Measurement of Visual Fields for Clinical Research: Guidelines From the Eye Care Technology Forum. Ophthalmology. 1996 Jan;103(1):186-9.
39. Imaging and Perimetry Society Standards and Guidelines. Optom Vis Sci. 2011 Jan;88(1):4-7.
40. Kerr NM, Chew SS, Eady EK, Gamble GD, Danesh-Meyer HV. Diagnostic accuracy of confrontation visual field tests. Neurology. 2010 Apr 13;74(15):1184-90.
41. Holmes JM, Leske DA Hohberger GG. Defining Real Change in Prism-Cover Test Measurements. Am J Ophthalmol. 2008 Feb; 145(2): 381–385.
42. de Jongh E, Leach C, Tjon-Fo-Sang MJ, Bjerre A. Inter-examiner Variability and Agreement of the Alternate Prism Cover Test (APCT) Measurements of Strabismus Performed by 4 Examiners. Strabismus. 2014 Dec;22(4):158-66.
43. Rowe FJ. Ocular alignment and motility: moving variables in clinical trials. Trans. 34th European Strabismological Association. Bruges, Belgium. 2011, pp. 111-114
44. Rowe FJ. Measurement of manifest deviations using corneal reflections. Optometry Today, 1994 ; Nov : 36-38
45. Chen HF, Wu CY, Lin KC, Chen CL, Huang PC, Hsieh CJ, Liu JS. Rasch validation of a combined measure of basic and extended daily life functioning after stroke. Neurorehabilitation and Neural Repair. 2013; 27: 125-32
46. Radner W. Reading charts in ophthalmology/ Graefe's Archives Clinical Experimental Ophthalmology. 2017; 255: 1465-1482
47. Trauzettel-Klosinski S, Dietz K, IReST Steering Group. Standardised assessment of reading performance: the New International Reading peed Texts IReST. Investigative Ophthalmology and Vision Science. 2012; 53: 5452-61
48. Sarker SJ, Rudd AG, Douiri A, Wolfe CD. Comparison of 2 extended activities of daily living scales with the Barthel Index and predictors of their outcomes: cohort study within the South London Stroke Register (SLSR). Stroke. 2012; 43: 1362-9
49. Green J, Forster A, Young J. A test-retest reliability study of the Barthel Index, the Rivermead Mobility Index, the Nottingham Extended Activities of Daily

For peer review only

Living Scale and the Frenchay Activities Index in stroke patients. Disability and Rehabilitation. 2001; 23: 670-6

50. Legg LA, Lewis SR, Schofield-Robinson OJ, Drummond A, Langhorne P. Occupational therapy for adults with problems in activities of daily living after stroke. Cochrane Database of Systematic Reviews 2017, Issue 7. Art. No.: CD003585. DOI: 10.1002/14651858.CD003585.pub3.

51. McKenna HP. The Delphi technique: a worthwhile research approach for nursing? Journal of Advanced Nursing. 1994; 19: 1221-25

For peer review only

Figures

Figure 1: Flow chart of COS and COM development process

Figure 2: Core outcome sets consensus

Figure 3: Core outcome measures consensus

For peer review only - http://bmjopen.bmj.com/site/about/guidelines.xhtml

Table 1, COS outcome extraction from overview of reviews

COM outcomes indicated in shaded cells

**** indicates COM domains***

Outcomes for COS	COS domains
Case history – open questions	Case history – open questions
Case history – specific questions<br>Eye strain, Reading difficulty, Blurred, altered or reduced vision, Visual field loss, Awareness of full environment, Oscillopsia, Diplopia, Polyopia, Visual hallucinations, Altered colour vision, Altered movement of objects, Depth perception misjudgements, Tilted images, Distorted images, Face /object recognition, Clutter difficulty, Getting lost, Prolongation of images, Reverse image size, Glare, Visual crowding, Visual disorientation	Case history – specific questions
Case history – carer open questions	Case history – carer open questions
Case history – carer specific questions<br>Personal care issues, Eyes constantly moving/jerking, Missing things to one side, Bumping into things, Concerns over vision, Visual hallucinations, Family/friend recognition, Difficulty naming objects, Getting lost, Reading problems	Case history – carer specific questions
Case history – previous ocular history	Case history – previous ocular history
Case history – glasses wear	Case history – glasses wear
Observations – open comments	Observations – open comments
Observations – specific features<br>Lids, Pupils, Squint – misaligned eyes, Eye movements, Turning head to see, Closing one eye to see better, Misjudging distances, Wobbling eyes	Observations – specific features
logMAR charts, Snellen charts, Fixation and following observation, Vanishing optotype charts, Grating charts, Near acuity charts, Kay's pictures, Sheridan Gardiner single optotypes, Lea symbols	Visual acuity*
Fundus check, Retinal photography/OCT	Ocular health*
Cover uncover test, Alternating cover test, Observations of corneal reflections	Eye alignment position*

For peer review only - http://bmjopen.bmj.com/site/about/guidelines.xhtml

Nine positions of gaze, Horizontal gaze only, Vertical gaze only, Horizontal and vertical gaze only, Vergence, Saccade movement, Smooth pursuit movement, Optokinetic nystagmus movement, Vestibulo-ocular reflex, Hess/Lees/Harms wall charts	Eye movement assessment*
Retinal correspondence, Sensory fusion, Motor fusion, Stereopsis	Binocular vision assessment*
Prism cover test, Krimsky test, Prism reflection test, Synoptophore, Bruckner test, Maddox rod	Eye alignment measurements*
Confrontation, Static central perimetry, Static peripheral perimetry, Kinetic perimetry	Visual field assessment*
Line bisection, Cancellation task – star, balloon, heart, etc., Clock drawing, Room/environment description, Behaviour inattention test battery	Visual neglect assessment*
Observed navigation, Reading, Eye scanning, Walking observations, Activities of daily living, Self-care, Body placement, Spatial awareness, Mobility observations, Writing, Hand-eye coordination, Visual memory and cognition, Visual perception	Functional assessment*
Special test, e.g. Wilkins, iRest, Radner, Newspaper/magazine, Book	Reading assessment*
Vision-related, e.g. VFQ25, DLDV; Health-related, e.g. SF12; Activity of daily living, e.g. IADL; Extended activity of daily living, e.g. NEADL	Questionnaires*
Visual perception – checklist	Visual perception – checklist*
Swinging flashlight test	Pupil assessment*
Palpebral apertures, Lid function test	Lid assessment*
Pelli-Robson chart, Mars test, VisTech	Contrast sensitivity assessment*
Ishihara test, City test	Colour vision assessment*

OCT – Optical Coherence Tomography; iReST - International Reading Speed Test; VFQ25 – Visual Function Questionnaire 25; DLDV – Daily Living tasks Dependent on Vision; SF12 – Short Form 12; IADL – Instrumental Activities of Daily Living; NEADL – Nottingham Extended Activities of Daily Living

For peer review only - http://bmjopen.bmj.com/site/about/guidelines.xhtml

Table 2, Quality indicators of included instruments

	**Visual acuity**	**Visual field**	**Ocular alignment**	**Ocular movement**	**Quality of life**	**Functional vision**
	LogMAR	***Peripheral perimetry***	***Prism cover test***	***Rotation grading***	***VFQ-25***	***Radner reading***
Use in target population	Yes	Yes	Yes	Yes	Yes	Yes
**Content validity**						
Reliability	+	+	+	+	+	+
Responsiveness	+	+	+	+	+	+
Internal consistency	+	+	+	+	+	+
Structural validity	+	+	+	+	+	+
Measurement error	+	+	+	+	+	+
Hypothesis testing	+	+	+	+	+	+
Criterion validity	+	+	+	+	+	+
Cross-cultural validity	+	+	+	+	+	+
**Quality of evidence**	High	High	Moderate	Moderate	Moderate	Moderate
**Feasibility**						
Patient comprehension	Widely understood	Widely understood	Widely understood	Widely understood	Widely understood	Widely understood
Interpretability	Clear usage	Clear usage	Clear usage	Clear usage	Clear usage	Clear usage
Ease of administration	High	Relative	High	High	High	High
Length of instrument	Small - small number of lines	Moderate – can take time per eye	Small	Small	Moderate	Moderate
Completion time	Within minutes	15-20 minutes	Within minutes	Within minutes	Relative	Relative
Patient mental ability level	Low – use with children	Medium	Low	Low	Medium	Medium
Ease of standardisation	High ease	High	High	High	Moderate	Moderate
Clinician comprehension	Widely understood	Widely understood	Widely understood	Widely understood	Widely understood	Widely understood

For peer review only - http://bmjopen.bmj.com/site/about/guidelines.xhtml

Type of instrument	Letter chart	Perimeter	Occluder, target, prisms	Occluder, target	Questionnaire	Reading paragraphs
Cost	Low	High	Low	Low	Relative to licence	Low
Required equipment	Letter chart	Perimeter	Occluder, target, prisms	Occluder, target	Questionnaire	Book
Type of administration	Clinician-led	Clinician-led	Clinician-led	Clinician-led	Clinician-led Self-administered	Clinician-led
Availability in different settings	Yes	Yes	Yes	Yes	Yes	Yes
Copyright	Per company	Per company	Per company	Per company	Per company	Per company
Patient physical ability level	Minimal	Relative	Minimal	Minimal	Relative	Relative
Regulatory approval	No	No	No	No	No	No
Ease of score calculation	High ease	High ease	High ease	High ease	High ease	High ease

For peer review only

For peer review only - http://bmjopen.bmj.com/site/about/guidelines.xhtml

Table 3, Reasons for exclusion of instruments

Instrument / measurement	Exclusions
Case history	Non-standardised and subjective
Observations	Non-standardised and subjective
Visual acuity (non-logMAR options), e.g.: Snellen charts; Fixation and following observation; Vanishing optotype charts; Grating charts; Near acuity charts; Kay's pictures; Sheridan Gardiner single optotypes; Lea symbols	Inconsistencies in measurement properties
Ocular position, e.g.: Krimsky test; Prism reflection test; Synoptophore; Bruckner test; Maddox rod	Inconsistencies in measurement properties and inclusion of subjective examiner judgments
Ocular movements, e.g.: Hess/Lees/Harms wall charts	Insufficient widespread access and insufficient assessment of peripheral eye movements
Visual fields: Confrontation; Central perimetry	Subjective examiner assessment and/or insufficient assessment of the peripheral field of vision beyond 30 degrees
Reading, e.g.: IReST; Wilkins; Vocational reading charts	Reading sections set at specific font size Non-adaptable to low vision patients
Activities of daily living, e.g.: ADL (Activities of Daily Living); AMPS (Assessment of Motor and Process Skills); FAI (Frenchay Activities Index); FIM (Functional Independence Measure); MET (Multiple Errands Test); RNLI (Reintegration to Normal Living Index); SIS (Stroke Impact Scale); LIFE-H (Assessment of Life Habits); ACS (Activity Card Sort); MRS (Modified Rankin Scale)	No one specific test targeting vision-related activities of daily living
Vision-related Quality of life, e.g.: VA LV (Veterans Low Vision Visual Function Questionnaire); AI (Activity Inventory); DLTV (Daily Living Tasks Dependent on Vision); SRA-VFP (Self-Reported Assessment of Functional Visual Performance)	Inconsistencies in measurement properties

For peer review only

For peer review only - http://bmjopen.bmj.com/site/about/guidelines.xhtml

Figure 1, Flowchart of COS and COM development process

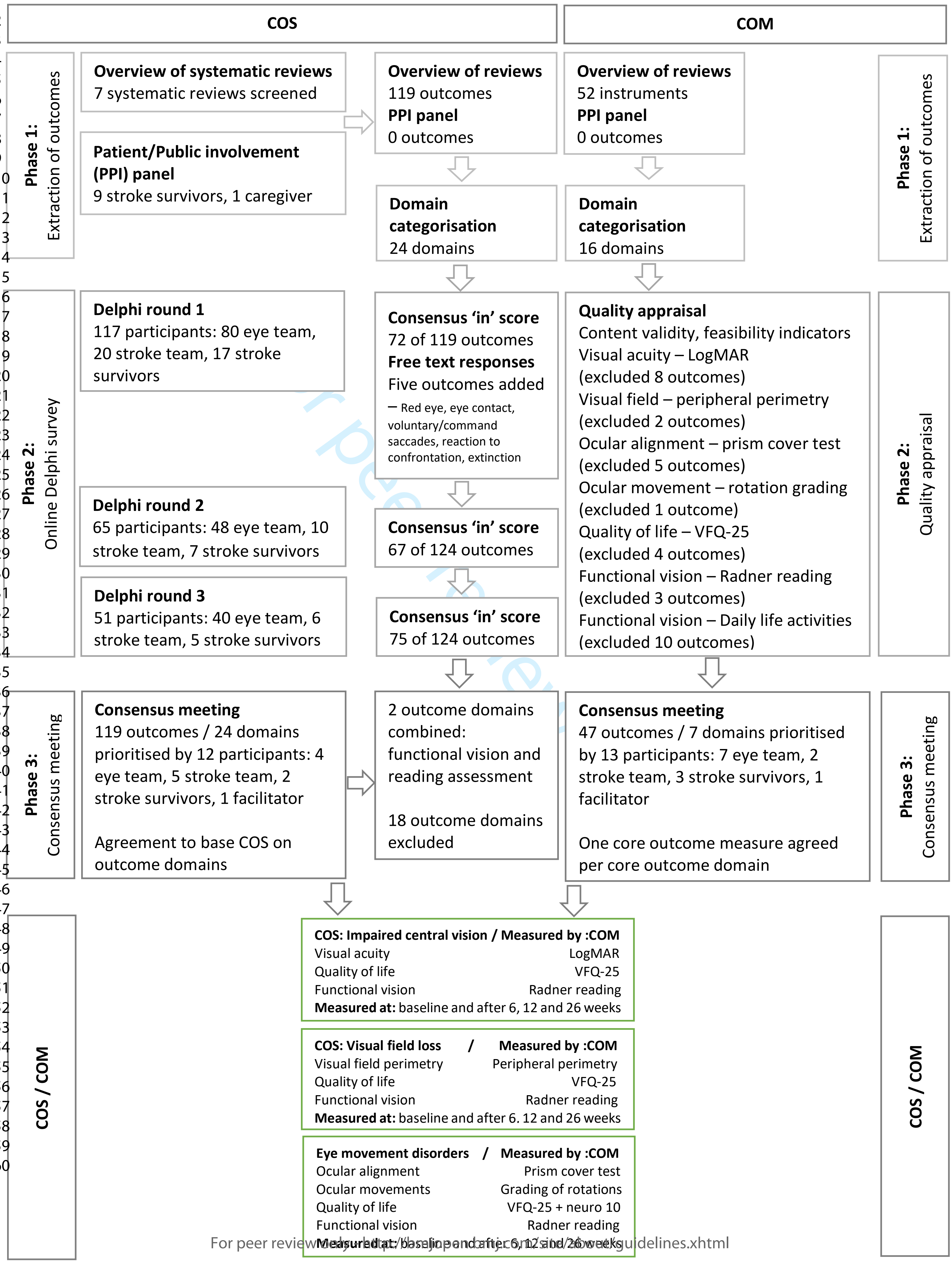

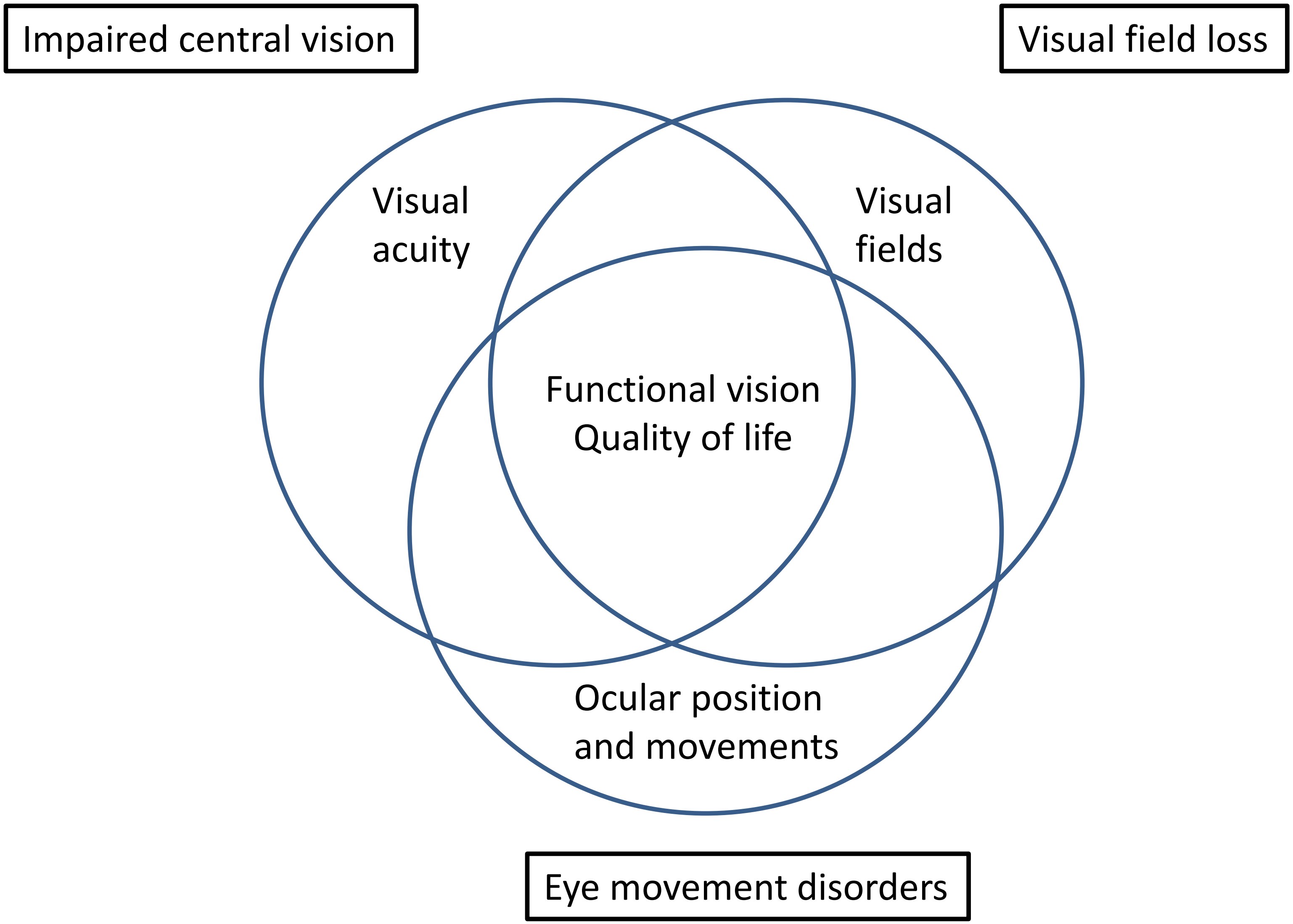
Impaired central vision
Visual field loss
Visual acuity
Visual fields
Functional vision
Quality of life
Ocular position and movements
Eye movement disorders

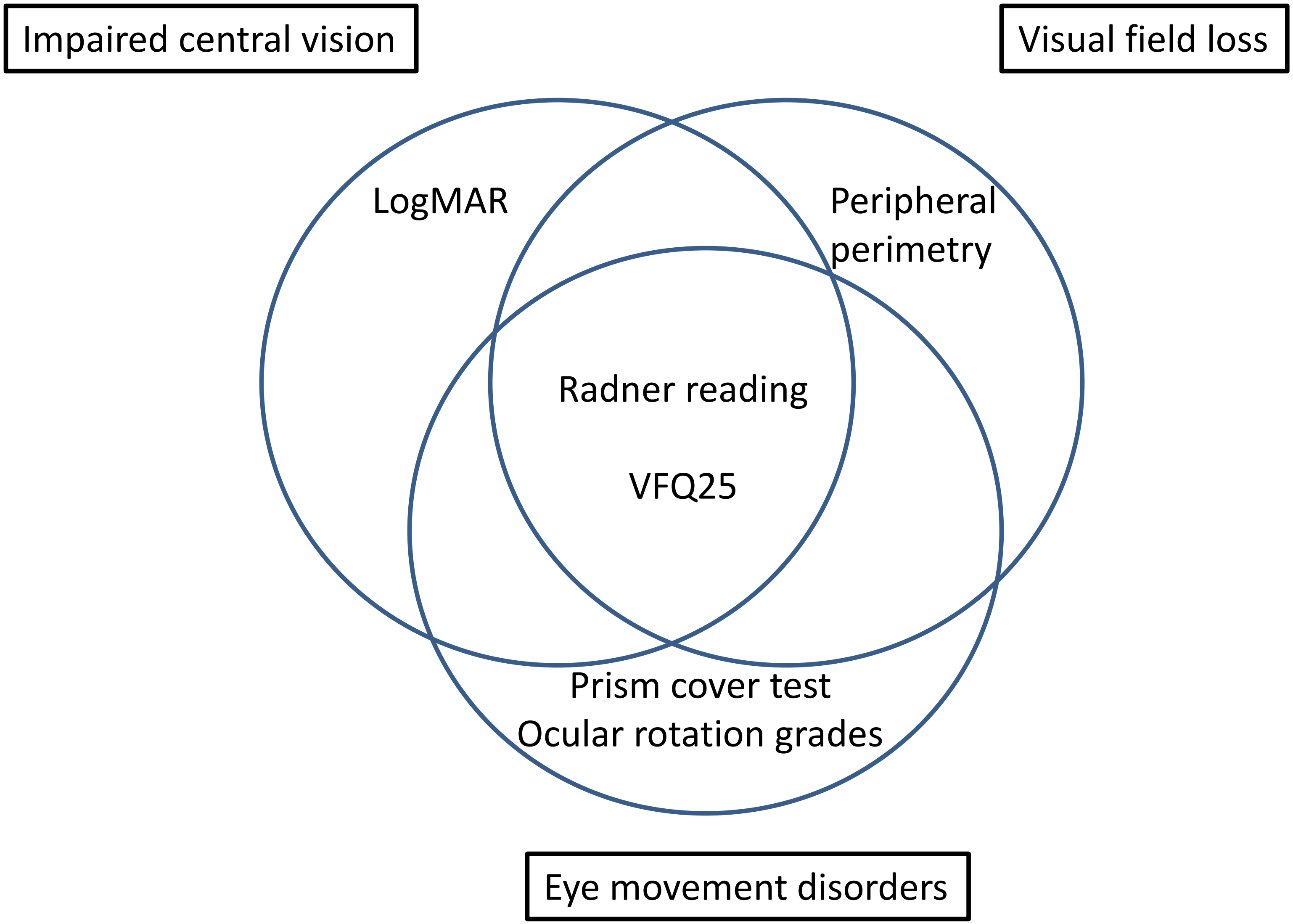
Impaired central vision
Visual field loss
LogMAR
Peripheral perimetry
Radner reading
VFQ25
Prism cover test
Ocular rotation grades
Eye movement disorders

COMET initiative registration

https://www.comet-initiative.org/Studies/Details/1420

Development of core outcome sets for vision screening and assessment in stroke: a Delphi and consensus study

OBJECTIVES: Visual impairment following stroke is common with a reported incidence of visual impairment in 60% of stroke survivors. Screening for visual impairment is neither routine nor standardised. This results in a health inequality where some stroke survivors receive comprehensive vision assessment to identify any existent visual problems while others receive no vision assessment leaving them with unmet needs from undiagnosed visual problems. The aim of this study was to define two core outcome sets (COS), one for vision screening and one for full visual assessment of stroke survivors. DESIGN: A list of potentially relevant visual assessments was created from a review of the literature. The consensus process consisted of an online 3-round Delphi survey followed by a consensus meeting of the key stakeholders. PARTICIPANTS: Stakeholders included orthoptists, occupational therapists, ophthalmologists, stroke survivors and COS users such as researchers, journal editors and guideline developers. SETTING: University. OUTCOME MEASURES: COS. RESULTS: Following the consensus process we recommend the following nine assessments for vision screening: case history, clinical observations of visual signs, visual acuity, eye alignment position, eye movement assessment, visual field assessment, visual neglect assessment, functional vision assessment and reading assessment. We recommend the following 11 assessments for full vision assessment: case history, observations, visual acuity, eye alignment position, eye movement assessment, binocular vision assessment, eye position measurement, visual field assessment, visual neglect assessment, functional vision assessment, reading assessment and quality of life questionnaires. CONCLUSIONS: COS are defined for vision screening and full vision assessment for stroke survivors. There is potential for their use in reducing heterogeneity in routine clinical practice and for improving standardisation and accuracy of vision assessment. Future research is required to evaluate the use of these COS and for further exploration of core outcome measures.

Aim
The aim of this study was to define two core outcome sets (COS), one for vision screening and one for full visual assessment of stroke survivors.

Contributors
Rowe, F. J. Hepworth, L. R. Kirkham, J. J.

Publication

Journal: BMJ Open
Volume: 9
Issue: 9
Pages: e029578 -
Year: 2019
DOI: 10.1136/bmjopen-2019-029578

Further Study Information

Current Stage: Not Applicable
Date:
Funding source(s): This article/paper/report presents independent research funded by the National Institute for Health Research (NIHR: CDF-2012-05-126).

Health Area

Disease Category: Eyes & vision, Neurology

Disease Name: Visual impairment after stroke

Target Population

Age Range: 18 - 100

Sex: Either

Nature of Intervention: Other, Screening

Stakeholders Involved

- Clinical experts
- Consumers (caregivers)
- Consumers (patients)
- Guideline developers
- Journal editors
- Researchers

Study Type

- COS for clinical trials or clinical research
- COS for practice

Method(s)

- Consensus meeting
- Delphi process
- Literature review

A list of potentially relevant visual assessments was created from a review of the literature. The consensus process consisted of an online 3-round Delphi survey followed by a consensus meeting of the key stakeholders.

only

Core Outcome Set-STandards for Reporting: The COS-STAR Statement Checklist

SECTION/TOPIC	ITEM No.	CHECKLIST ITEM	REPORTED ON PAGE NUMBER
TITLE/ABSTRACT			
Title	1a	Identify in the title that the paper reports the development of a COS	1
Abstract	1b	Provide a structured summary	2
INTRODUCTION			
Background and Objectives	2a	Describe the background and explain the rationale for developing the COS.	4
	2b	Describe the specific objectives with reference to developing a COS.	4
Scope	3a	Describe the health condition(s) and population(s) covered by the COS.	4
	3b	Describe the intervention(s) covered by the COS.	4
	3c	Describe the setting(s) in which the COS is to be applied.	4-5
METHODS			
Protocol/Registry Entry	4	Indicate where the COS development protocol can be accessed, if available, and/or the study registration details.	5
Participants	5	Describe the rationale for stakeholder groups involved in the COS development process, eligibility criteria for participants from each group, and a description of how the individuals involved were identified.	5, 6, 8, 9
Information Sources	6a	Describe the information sources used to identify an initial list of outcomes.	6, 9
	6b	Describe how outcomes were dropped/combined, with reasons (if applicable).	7, 8, 9
Consensus Process	7	Describe how the consensus process was undertaken.	8, 9
Outcome Scoring	8	Describe how outcomes were scored and how scores were summarised.	7, 8, 9
Consensus Definition	9a	Describe the consensus definition.	7, 9
	9b	Describe the procedure for determining how outcomes were included or excluded from consideration during the consensus process.	7, 8, 9
Ethics and Consent	10	Provide a statement regarding the ethics and consent issues for the study.	5, 18
RESULTS			
Protocol Deviations	11	Describe any changes from the protocol (if applicable), with reasons, and describe what impact these changes have on the results.	N/A
Participants	12	Present data on the number and relevant characteristics of the people involved at all stages of COS development.	10, 11, 12

For peer review only

Outcomes	13a	List all outcomes considered at the start of the consensus process.	10, 11, 12
	13b	Describe any new outcomes introduced and any outcomes dropped, with reasons, during the consensus process.	11, 12, 13, 14
COS	14	List the outcomes in the final COS.	13
DISCUSSION			
Limitations	15	Discuss any limitations in the COS development process.	15, 16
Conclusions	16	Provide an interpretation of the final COS in the context of other evidence, and implications for future research.	17
OTHER INFORMATION			
Funding	17	Describe sources of funding/role of funders.	17
Conflicts of Interest	18	Describe any conflicts of interest within the study team and how these were managed.	17

From: Kirkham JJ, Gorst S, Altman DG, Blazeby JM, Clarke M, Devane D, et al. (2016) Core Outcome Set–STAndards for Reporting: The COS-STAR Statement. PLoS Med 13(10): e1002148. https://doi.org/10.1371/journal.pmed.1002148

For peer review only

BMJ Open

Development of core outcome sets and core outcome measures for central visual impairment, visual field loss and ocular motility disorders due to stroke: a Delphi and consensus study.

Journal:	*BMJ Open*
Manuscript ID	bmjopen-2021-056792.R2
Article Type:	Original research
Date Submitted by the Author:	13-Feb-2022
Complete List of Authors:	Rowe, Fiona; University of Liverpool<br>Hepworth, Lauren; University of Liverpool, Health Services Research<br>Kirkham, Jamie J.; Manchester University, Biostatistics
**Primary Subject Heading**:	Ophthalmology
Secondary Subject Heading:	Health services research
Keywords:	Stroke < NEUROLOGY, STROKE MEDICINE, OPHTHALMOLOGY, Neuro-ophthalmology < OPHTHALMOLOGY

SCHOLARONE™
Manuscripts

I, the Submitting Author has the right to grant and does grant on behalf of all authors of the Work (as defined in the below author licence), an exclusive licence and/or a non-exclusive licence for contributions from authors who are: i) UK Crown employees; ii) where BMJ has agreed a CC-BY licence shall apply, and/or iii) in accordance with the terms applicable for US Federal Government officers or employees acting as part of their official duties; on a worldwide, perpetual, irrevocable, royalty-free basis to BMJ Publishing Group Ltd ("BMJ") its licensees and where the relevant Journal is co-owned by BMJ to the co-owners of the Journal, to publish the Work in this journal and any other BMJ products and to exploit all rights, as set out in our licence.

The Submitting Author accepts and understands that any supply made under these terms is made by BMJ to the Submitting Author unless you are acting as an employee on behalf of your employer or a postgraduate student of an affiliated institution which is paying any applicable article publishing charge ("APC") for Open Access articles. Where the Submitting Author wishes to make the Work available on an Open Access basis (and intends to pay the relevant APC), the terms of reuse of such Open Access shall be governed by a Creative Commons licence – details of these licences and which Creative Commons licence will apply to this Work are set out in our licence referred to above.

Other than as permitted in any relevant BMJ Author's Self Archiving Policies, I confirm this Work has not been accepted for publication elsewhere, is not being considered for publication elsewhere and does not duplicate material already published. I confirm all authors consent to publication of this Work and authorise the granting of this licence.

For peer review only

Development of core outcome sets and core outcome measures for central visual impairment, visual field loss and ocular motility disorders due to stroke: a Delphi and consensus study.

Fiona J Rowe[1], PhD, Lauren R Hepworth[1], PhD, Jamie J Kirkham[2], PhD

1, Institute of Population Health, University of Liverpool, UK

2, Centre for Biostatistics, The University of Manchester, Manchester Academic Health Science Centre, Manchester, UK

Address for correspondence:

Prof Fiona Rowe

Waterhouse Building Block B,

University of Liverpool,

1-3 Brownlow Street,

Liverpool L69 3GL

T: 0151 7944956

E: rowef@liverpool.ac.uk

Twitter: @orthopticbrain

Category: Original research article

Short title: Core outcomes for vision and stroke

Word count: 4542

Number of tables: 3

Number of figures: 3

Supplementary data: 2

For peer review only

Abstract

Objectives: Reporting of research for stroke-related visual impairment is inconsistent. The aim of this study was to define three core outcome sets (COS) and related core outcome measurements (COM) for central visual impairment, visual field loss and ocular motility disorders in stroke research.

Design: The consensus process consisted of an online 3-round Delphi survey followed by a consensus meeting of key stakeholders.

Setting: UK-wide survey.

Participants: Stakeholders included orthoptists, occupational therapists, ophthalmologists, stroke survivors and COS users such as researchers, journal editors and guideline developers.

Outcome measures: For COS development, a list of potentially relevant visual outcomes was created after review of the literature and further grouped into outcome domains. For COM development, a list of potential instruments was created after review of the literature and quality appraised for reliability and validity.

Results: COS - 119 potential outcomes extracted from published literature. Similar assessment outcomes were grouped into 24 outcome domains. Delphi process included 123 participants in round 1, 65 round 2, 51 round 3. Twelve participants attended the consensus meeting with recommended outcome domains for central visual impairment (visual acuity, functional vision, quality of life), visual field loss (visual fields, functional vision, quality of life) and ocular motility disorders (eye alignment, eye movements, functional vision, quality of life). COM - 52 test options extracted from the COS outcomes and grouped into 16 domains. Thirteen participants attended the COM consensus meeting. Recommended instruments for measurement of these outcomes include; LogMAR visual acuity, cover test, cardinal position eye movement assessments, peripheral visual field perimetry, Visual Function Questionnaire-25.

Conclusions: COS and COM are defined for vision research for stroke survivors. Their use has potential to reduce heterogeneity in routine clinical practice and

For peer review only

For peer review only - http://bmjopen.bmj.com/site/about/guidelines.xhtml

improve standardisation and accuracy of vision assessment. Future research is required to evaluate the use of these COS and COM.

Keywords: Core outcome set; Core outcome measures; Vision; Stroke; Delphi; Consensus; Central vision; Visual field loss; Ocular motility

Strengths and limitations of this study

- We report a new core outcome set (COS) and core outcome measures (COM) for stroke-vision research.
- The COS and COM have a break-down for category of visual impairment; specifically, central vision impairment, visual field loss and ocular motility disorders.
- Composite outcome domains were ranked by Delphi and consensus opinion from multi-disciplinary experts and stroke survivors.
- This was not an international study; participants were from UK and Ireland.
- We experienced attrition bias across the Delphi rounds.

For peer review only

Introduction

Visual impairment is common as a consequence to stroke with a reported incidence of 60% in stroke survivors [1]. Types of visual impairment generally fall into one of four categories including impairments of central vision, eye movements, visual fields and visual perception [2]. The impact of visual impairment on stroke survivors is wide reaching. Visual impairment can increase risk of collisions and falls, impede activities of daily life such as reading and writing, prevent return to work, pre-stroke activities and hobbies, and driving [3-6]. Such impacts result in loss of independence, increase likelihood of social isolation and results in mood change, anxiety and depression [5,6]. There are several approaches to the management of these visual impairments including visual scanning therapy, prisms, occlusion, spectacles, drugs, surgery, botulinum toxin, exercises, or a combination of two or more of the above [7-9].

A range of systematic reviews in the field of stroke-vision research have identified that there is considerable variation in the outcomes being measured and reported in primary research studies, which impacts the ability to compare and synthesise outcome results across studies. Moreover, it was noted that there is a paucity of outcome data available on important patient outcomes such as quality of life and functional visual assessment [7-11].

To mitigate these issues and to increase the relevance of research, a core outcome set (COS) and core outcome measurements (COM) can be developed which represents an agreed standardised set of outcomes and instruments that should be used and reported in all studies for a specific area of health or healthcare [12,13]. A search of the COMET (Core Outcome Measures in Effectiveness Trials) database revealed that there are several studies that have investigated important outcomes for eyes and vision disease; examples include cataracts and glaucoma but none has specifically looked at stroke [14].

The aim of this study was to achieve consensus on the content of vision research outcomes for stroke survivors. In this study we report the results of a Delphi process and consensus meetings in the development of three COS and COM for stroke-

For peer review only - http://bmjopen.bmj.com/site/about/guidelines.xhtml

vision research for the categories of impaired central vision, visual field loss and ocular motility disorders.

Methods

Ethical approval

This study had institutional ethical approval (Ref-1415-040) and was undertaken in accordance with the Tenets of Helsinki. Informed consent was obtained for the Delphi survey from all participants in the consensus meeting.

Development of the COS involved three phases: (1) the generation of a comprehensive list of outcomes; (2) a Delphi survey involving three rounds to gain consensus as to which outcomes are most important; and (3) patient and professional consensus meetings to agree on a final COS.

Development of the COM involved three phases: (1) the generation of a comprehensive list of outcome measurement instruments specific to the outcomes in each COS, (2) quality appraisal of the outcome measurement instruments and (3) a consensus process on recommendations for the selection of outcome measurement instruments.

A protocol for the development of this COS project was written by the steering committee, registered in the COMET initiative website (http://www.comet-initiative.org/studies/details/275?result=true) and available as open access (http://pcwww.liv.ac.uk/~rowef/index_files/Page356.html - supplementary file). When developing this COS, we followed the minimum set of development standards set out by COS-STAD (Core Outcome Set-STAndards for Development) Statement and COSMIN (Consensus-based Standards for the selection of health Measurement Instruments), and report the results against the COS-STAR (Core Outcome Set–STAndards for Reporting) guideline [13,15,16].

The full methods for the COS have been fully outlined previously [17] and detail the steering group, patient and public involvement, and stakeholder groups.

Patient and public involvement

For peer review only - http://bmjopen.bmj.com/site/about/guidelines.xhtml

This study was supported by a patient advisory group (the VISable patient and public involvement panel) which provided input to this research study. The VISable panel met on a regular basis during the conduct of the study. Patients contributed to the design of the study and were involved at all stages of the survey and consensus meeting.

Core outcome set

Phase 1 – Outcome identification

A literature review was conducted to develop a preliminary list of outcomes for the Delphi survey. We undertook an overview of seven systematic reviews of studies/trials reporting vision screening, assessment and treatment of post-stroke visual impairment [2,7-11,18-20] and extracted a list of included outcomes. The list was then circulated to the VISable patient and public involvement panel [21] for approval and checking of writing for lay terms as the basis of the online survey development. VISable (a panel of ten patients/carers) were asked to consider whether any further outcomes should be added, particularly those deemed relevant to patient-reported outcomes. The panel advised that appropriate patient-important outcomes were included already in the list and did not add any further outcomes.

Phase 2 – Delphi survey

We undertook a prospective consensus study using a Delphi process. SurveyMonkey (SurveyMonkey Inc. Palo Alto, California, USA 2015) was used as the online platform to administer the Delphi process. The survey was piloted for checks of ambiguity and appropriate use of lay language by the steering group and then released live. The full survey was circulated through professional, clinical and research networks in the UK and Ireland to reach stakeholder groups of eye care teams (orthoptists, ophthalmologists), stroke teams (physicians, nurses, therapists) and patients (stroke survivors).

The Delphi survey consisted of three rounds. Rounds remained open for 10 weeks, with regular two-week reminders sent to those that had partially completed or not completed the survey in order to maximise response rates. All terms had explanatory

For peer review only - http://bmjopen.bmj.com/site/about/guidelines.xhtml

notes to aid interpretation. All participants were asked to score each outcome in terms of importance and asked to identify any additional outcomes of importance that did not appear in the list of assessments. Additional outcomes added in round 1 were reviewed by the steering group to consider their relevance to the survey and to identify and remove duplicates.

Scoring Method: In each round, participants were asked to score the importance of each outcome listed on a nine-point scale (1-3: not important; 4-6: important but not critical; 7-9: critical) as well as an 'unable to score' option. The scale was devised by the GRADE working group [22] to score the quality of evidence for outcomes in systematic reviews and has been adopted in other core outcome set development work research using Delphi methods.

Methods of analysis: For each round of Delphi, the results for each outcome were summarised, which included providing the number of participants responding to the survey, as well as the mean, standard deviation and the percentage of participants scoring the outcome at each possible level from 1-9.

In round 2, all data from round 1 was analysed and compiled by stakeholder groups;1) stroke survivors/carers, 2) stroke team professionals and 3) eye team professionals, to allow different perspectives to be considered prior to re-rating [12]. The summarised results for each of the three stakeholder groups were provided to all participants in the form of histogram plots. Each participant was also shown their personal original score for each outcome. Participants had the opportunity to re-score based on the summary scores from the three stakeholder groups versus their previous personal scores. All participants were also asked to score on additional outcomes that were identified and added in round 1.

In round 3, the results from round 2 were analysed. Following analysis, the results from each stakeholder group were reviewed by the steering group and judged to be similar in terms of mean responses and percentage spread across the responses of 1 to 9. Thus, they were presented as one compilation of all responses. Each participant was shown their personal score from round 2 for each outcome along with the summary scores from all other participants, and asked to score each outcome again in terms of importance.

Consensus was defined *'a priori'* however, this information was not provided on the Delphi survey. Participants were aware of these cut off values at the time of

For peer review only - http://bmjopen.bmj.com/site/about/guidelines.xhtml

attending the consensus meeting and these values were applied overall to responses: 'Consensus in' (i.e. consensus that the outcome should be included in the core set) was defined as greater than 70% of participants scoring as 7-9 and less than 15% of participants scoring 1-3. 'Consensus out' (i.e. consensus that the outcome should not be included in the core set) was defined as greater than 70% of participants scoring as 1-3 and less than 15% of participants scoring as 7-9. All other combinations were seen as equivocal. The outcomes that were designated as 'consensus in' or seen as 'equivocal' were taken forward and discussed in more detail at the consensus meeting for inclusion into the final core outcome sets. Participants were reminded of all outcomes not reaching consensus as part of the Delphi process.

Phase 3 – Consensus meeting

All participants from the Delphi survey were invited to contribute to the consensus meeting. Those expressing interest were invited to attend a face to face consensus meeting. All round 3 survey completers were emailed an invitation. Our intention was to ensure all stakeholders were reasonably represented. The format of the meeting included a short study overview which outlined the categories of visual impairment due to stroke and outcomes identified from the outcome identification phase, a presentation containing a summary of the results and number of outcomes reaching consensus from the survey. Each outcome was to be considered in turn, in order of their presentation in the Delphi survey, to ratify these results. Each remaining outcome was then be considered in turn with full discussion. Similar to the survey, each outcome was considered as reaching consensus with 70% of participants voting in favour of its inclusion.

Each participant voted on every outcome being asked to vote 'Yes' (this outcome should be included in the COS), 'No' (this outcome should not be included), or 'Unsure' using voting slips. Results were collated after voting for all outcomes was completed and results then presented to the group. Outcomes were retained or dropped when consensus is reached. Discussion and further rounds of voting, restricting the options to 'Yes' or 'No' was undertaken until consensus was reached on all outcomes. All outcomes retained were to be included in the final COS.

For peer review only

For peer review only - http://bmjopen.bmj.com/site/about/guidelines.xhtml

Core outcome measures

Phase 1 – Outcome identification

From the same literature review as the COS, a comprehensive list of outcome measurement instruments was generated, with circulation of the list to the VISable panel for review.

Phase 2 – Quality appraisal

We undertook a quality appraisal of the available outcome measurements identified from the literature review. For each outcome measurement instrument, we conducted a further review of the medical literature to seek evidence of the use of the instrument in a stroke population. We evaluated the validity of the instrument and feasibility for its use with stroke survivors as per COSMIN guidance [13]. Measurement properties included content validity, internal structure, reliability, measurement error, hypotheses testing, cross-culture validity, criterion validity and responsiveness. Feasibility aspects included comprehensibility, interpretability, ease and type of administration, length of the instrument, completion time, patient mental and physical ability level, ease of standardisation and scoring, type of instrument, cost, equipment required, availability in different settings, copyright, and regulatory approval requirements. Quality of evidence was graded from low to high.

Phase 3 – Consensus meeting

A subsequent (and separate to COS meeting) consensus meeting was planned for discussion of the COM development. Representatives from the COS Delphi survey were invited to a face to face consensus meeting; all round 3 survey completers were emailed an invitation. The intention was to ensure all stakeholders were reasonably represented. The same process as outlined above for COS development was followed for introduction to the process, definition of consensus, voting, and compilation of results. The plan was to seek consensus agreement on the inclusion of one instrument for each category of stroke-visual impairment research; specifically, categories of impaired central vision, ocular motility disorders and visual field loss. Consensus agreement was only sought for the agreed COS outcomes. Further, consensus agreement was sought on the timing of measurement.

For peer review only

Results

Figure 1 outlines a flowchart of the results for number of participants and number of outcomes in the development and agreement of the COS and COM.

Core outcome set process

Phase 1 - Outcome identification

We extracted 119 outcomes, many of which were variations on assessments for specific visual functions. For the purposes of clarity during COS development, similar assessments were also combined into outcome domains. This process produced a list of 24 domains (Table 1).

Phase 2 – Delphi survey

In total 123 participants registered for round 1 of the Delphi survey. Six registered but did not answer any survey questions. The remaining participants comprised of 79 orthoptists, 20 occupational therapists (stroke team stakeholder group), 17 stroke survivors (stroke survivor/carer stakeholder group) and 1 ophthalmologist. The orthoptists and ophthalmologist formed the eye team stakeholder group. There were 20 males and 97 females with ages ranging from 18-84 years. In round 2, 65 participants completed the survey – an attrition of 44.5% from round 1. These participants comprised 47 orthoptists, 10 occupational therapists, 7 stroke survivors and 1 ophthalmologist. In round 3, 51 participants completed the survey – an attrition of 56.4% from round 1 (21.5% from round 2). These participants comprised of 39 orthoptists, 6 occupational therapists, 5 stroke survivors and 1 ophthalmologist.

Following completion of the survey of 119 outcomes in round 1, five additional outcomes were put forward by the participants for inclusion in round 2: red eye, eye contact, reaction to confrontation, visual extinction and voluntary/command saccades. Consensus 'in' scores were achieved for 72 outcomes. No outcomes scored less than 3. After round 2 of 124 outcomes, no new outcomes were introduced. Consensus 'in' scores were achieved for 67 outcomes. No outcomes scored less than 3. Following round 3 of 124 outcomes, consensus 'in' scores were achieved for 75 outcomes. The Delphi survey reached the following consensus:

For peer review only - http://bmjopen.bmj.com/site/about/guidelines.xhtml

- Full consensus for inclusion was obtained for case history asking patients/carers open and specific questions plus checks of previous ocular history and glasses wear. Full consensus for observations, tests of visual acuity, eye alignment, eye movements, binocular vision, alignment measurement, visual fields, visual inattention, reading, lids and pupils, visual perception and functional visual assessment.
- No consensus was reached for eye contact during conversations, use of questionnaires, contrast sensitivity, colour vision, fundus checks and use of retinal photography/optical coherence tomography.

Phase 3 – Consensus meeting

The consensus meeting was a 1-day event held in Liverpool, UK with 12 participants comprising 5 occupational therapists (2 with research roles), 3 orthoptists (1 with a research role), 2 patients, 1 Cochrane editor and 1 facilitator. The facilitator did not take part in the voting. The objective of the meeting was to discuss and vote on the Delphi outcomes – the results of the Delphi survey had been provided to participants prior to and during the meeting.

During the consensus meeting, final consensus for research vision assessment was split according to category of visual impairment, (impaired central vision, visual field loss and ocular motility disorders). Participants decided to organise the COS on outcome domains rather than specific outcomes on the basis that many outcomes were similar and were better represented as a grouped outcome domain (see grouping of outcomes in Table 1). Two outcome domains were combined because of overlap in outcomes: functional vision and reading assessments.

No consensus could be reached for visual inattention or for visual perceptual disorders. Visual perceptual disorders were considered too heterogenous with so many different types of visual perception issue occurring. It was therefore not possible to determine a core set of assessments. Visual inattention was considered too broad and variable a condition with a discussion amongst consensus meeting participants about context and timing of assessment for stroke survivors with visual attention. Agreement was therefore obtained only for categories of impaired central vision, visual field loss and eye movement disorders (Figure 2). Impaired central vision consisted of visual acuity, functional vision and quality of life. Visual field loss

For peer review only - http://bmjopen.bmj.com/site/about/guidelines.xhtml

For peer review only

consisted of visual field perimetry, functional vision and quality of life. Eye movement disorders consisted of ocular alignment, ocular movement, functional vision and quality of life.

Core outcome measures process

Phase 1 - Outcome identification

From 119 outcomes identified for the COS, we extracted 52 test options. For the purposes of clarity, similar tests were combined into outcome domains. This process produced a list of 16 domains (Table 1).

Phase 2 – Quality appraisal

Quality assessment was conducted for the available outcome measurement instruments considering their measurement properties and feasibility aspects. Table 2 outlines the appraisal summary for selected instruments meeting quality indicators specific to the outcomes selected for the three COS of central vision impairment, visual field loss and ocular motility disorders; Logarithm of the Minimal Angle of Resolution (LogMAR) visual acuity, peripheral perimetry, prism cover test, ocular rotation grading, vision-related quality of life and reading.

Phase 3 – Consensus meeting

The consensus meeting was held as an online event with 13 participants comprising 2 occupational therapists, 7 orthoptists (2 with a research role), 3 patients and 1 facilitator. The facilitator did not take part in the voting. The objective of the meeting was to discuss and vote on the COM – the results of a comprehensive list of outcome measurements and their quality appraisal had been provided to participants prior to and during the meeting. Each participant was invited to make queries for each instrument and seek clarification of any part of the quality appraisal. Agreement was requested from each participant on inclusion of each selected instrument.

During the consensus meeting, final consensus for research vision assessment was split according to type of measurement (Figure 3). Overall excluded instruments and reasons for exclusion are outlined in table 3. Recommendation for inclusion was obtained for;

For peer review only - http://bmjopen.bmj.com/site/about/guidelines.xhtml

- LogMAR test (impaired central vision). This test may require the use of a matching card because of communication or cognitive issues.
- Peripheral perimetry (visual field loss). Specifically, peripheral visual field testing was recommended over central visual field testing to acknowledge the need to detect presence of peripheral visual field loss outside the central 30 degrees and detection of change occurring in the peripheral visual field over time. Kinetic perimetry was recommended with the additional of static points to check the integrity of the visual field within the peripheral boundary. Where static-only programmes were available, a recommendation was made to use the Esterman programme which is most commonly available regardless of equipment type. A recommendation was made to use the binocular Esterman programme as the minimum core assessment but with the addition of monocular Esterman fields for each eye where possible dependent on patient ability.
- Prism cover test and ocular rotation grading (ocular movement disorders). Ocular alignment determined by the cover test and measured by prism cover test. Ocular rotation gradings specifically in nine positions of gaze and assessed using saccadic and smooth pursuit movements alongside vergence (convergence and divergence). Recommendation to incorporate vestibulo-ocular reflex movements and/or optokinetic nystagmus where relevant (e.g. if smooth pursuit movements cannot be fully examined).
- VFQ-25 questionnaire (vision-related quality of life) [23]. Recommendation to add neuro-10 supplement when used for ocular motility COS but not appropriate for use in impaired central vision or visual field loss COS.
- Radner test (functional vision: reading impairment) [24]. Recommendation to capture additional impact to reading by non-visual issues such as presence of aphasia.

No consensus could be reached for functional vision assessment incorporating activities of daily living. Functional visual assessment was considered too heterogenous for types of assessment in relation to visual function versus general function and therefore it was not possible to determine one, or even a small number, of measurement tools. Consensus was reached that the aspect of functional vision with respect to activities of daily living warrants further research and should consider

For peer review only - http://bmjopen.bmj.com/site/about/guidelines.xhtml

the issues identified during the Delphi process and focus groups, including; observed navigation, eye scanning, walking observations, activities of daily living, self-care, body placement, spatial awareness, mobility observations, writing, hand-eye coordination, visual memory and cognition and visual perception.

The final stage of the consensus meeting was to consider the time points at which the COM should be made as a minimum. Consensus was achieved for a recommended measurement timescale of baseline and follow-up periods of 4 weeks, 12 weeks and 26 weeks, for all three COS. There was a recommendation that consideration should be given to recruitment in acute versus chronic stages of stroke given that rapid changes may occur in visual function within the first month of stroke onset.

Discussion

A core outcome set (COS) is an agreed minimum set of outcome measures that should be reported. By reporting a minimum set of measures, this reduces the heterogeneity of outcomes across studies, which in turn supports future evidence synthesis [12,15,16]. Once a COS is defined, it is important to achieve consensus on how the outcomes should be measured [13]. Core outcome measurements (COM) define how to measure core outcomes and are the instruments selected to measure each core outcome [13].

We have previously used Delphi and consensus process methods to develop test batteries for general clinical vision screening and assessment for post-stroke visual impairment [17]. The purpose of this present study was to agree on a COS and COM specific to use in stroke-vision research, distinct from routine clinical screening and assessment practice. After round 3 and the consensus meeting, stakeholders agreed with the proposed COS for vision research for three categories of visual impairment, consisting of three outcome domains for impaired central vision, three for visual field loss and four for eye movement disorders. Core amongst all categories were the domains of functional vision and quality of life. It is important to acknowledge these COS do not exclude further visual assessments which should be added as appropriate and relevant for the individual research study. Indeed, it is essential to

For peer review only - http://bmjopen.bmj.com/site/about/guidelines.xhtml

ensure that the multiple visual morbidities that arise due to stroke, are also properly recognised, sought and reported, despite not being selected as core elements.

Generally, one instrument is selected for each COM [13]. For assessment of impaired central vision, the selected instrument to test visual acuity was a logMAR chart, for visual field loss, peripheral perimetry was selected and for eye movement disorders, the selected instrument was the prism cover test when measuring ocular alignment, and grading of ocular rotations for measuring eye movement. For assessment of vision-related quality of life, the VFQ-25 questionnaire was selected and for functional vision assessment specific to reading, the Radner reading test was selected. It was not possible to reach consensus for measurement of other aspects of functional vision. There was unanimous agreement that further research is required to evaluate measurements of activities of daily living in relation to vision versus general function with specific aspects to be considered such as observed navigation and mobility, spatial awareness, self-care and writing. Quality appraisal for COM considered documentation of the use of the instrument in the target population, content validity, quality of evidence, and feasibility of use of the instrument [25-50]. Timing of these measurements were recommended at minimum time points of baseline and follow-up at 4, 12 and 26 weeks.

There are a number of strengths for this study. A COS has been produced for stroke-vision research with a break-down for category of visual impairment; specifically, central vision impairment, visual field loss and ocular motility disorders. This addresses a gap in evidence-based practice for post-stroke visual impairment. The COS is composed of outcome domains which were ranked by Delphi and consensus opinion from multi-disciplinary experts and stroke survivors. Furthermore, they are based on existent outcomes for which there are assessment instruments (i.e. COM) that are easily accessible for implementation, and accepted as validated clinical measures. Although these COS and COM were developed for stroke, there is potential for their use with other types of acquired brain injury causing visual impairment.

There are limitations to this study. Despite circulation of the survey across a wide range of networks, not all members of eye care and stroke teams took part despite invitation, e.g. lack of optometry, neuropsychology input, ophthalmology / neuro-

For peer review only - http://bmjopen.bmj.com/site/about/guidelines.xhtml

ophthalmology. Participants were from UK and Ireland and largely represented views and practices within the National Health Service (NHS). Thus, assessments are those used clinically in these countries. However, we are aware that the assessment outcome domains selected for the COS and instruments selected for the COM are those that are widely used internationally in clinical and research settings as evidenced from our initial literature review [2,8,10]. Indeed, the quality appraisal of outcome measurement instruments involved many studies undertaken in countries other than the UK [25-50]. We experienced attrition bias across the Delphi rounds. The attrition rate at round 2 was 44.5% and, by round 3, was 56.4% from the initial sample. High attrition rates however are common using Delphi methods and our rates can be considered within an acceptable range based on those reported for other COS developments [51]. Nevertheless a larger sample would have been preferable. Researcher bias is also a potential limitation. We aimed to limit this by providing a summary of results across all rounds of the Delphi survey and with final decisions left to the consensus meeting with experts. There can be risks from using Delphi methods in which participants can have very disparate views of each outcome. However, we sought a wide variety of participants across a number of stakeholder groups to achieve greater consistency in responses and balance potential outlier responses. Further, this core outcome development process included a final stage of consensus meetings such that decisions were not purely made from the Delphi responses. Although we initially included outcomes for visual neglect/inattention and visual perception, it was not possible to reach consensus for a COS for these outcomes through the Delphi survey or consensus process. A detailed discussion took place during the consensus process that highlighted the specific issues for these outcomes and, in particular, the issue of context of assessment (where, when) and type (personal/peri-personal/extra-personal) of visual neglect, and significance of heterogeneity for forms of other visual perceptual disorders. Further it was not possible to reach consensus for a COM for functional vision specific to activities of daily living. Discussion during the consensus meeting highlighted that no specific instrument/tool for activities of daily living focussed on functional vision and that this represented a gap in evidence requiring further research. Whilst visual inattention, visual perception disorders and functional visual assessment were not included in the COS and COM developed in this study, these are topics that warrant further research to target core outcomes and measurements.

For peer review only - http://bmjopen.bmj.com/site/about/guidelines.xhtml

These disorders can be debilitating consequences of stroke affecting the visual regions of the brain and we advocate for further research on development of COS and COM as being essential for these disorders.

Conclusions

This study reports the use of Delphi and consensus methods in the development of a core outcome set and core outcome measurements for stroke-vision research. Impaired central vision and visual field loss each comprised three outcome domains and eye movement disorders comprised four outcome domains. Each COS outcome domain has one recommended outcome measurement instrument except for functional vision relating to activities of daily living for which no consensus could be reached. These core outcome sets and core outcome measurements will facilitate standardisation of stroke-vision assessment in order to reduce heterogeneity in assessment in future research. Further research is now required to evaluate the use of these core outcome sets and outcome measures.

Acknowledgements

We thank all participants involved in the Delphi survey and consensus meetings. We also thank our clinical and patient colleagues who contributed to the study steering group.

Declarations of interest

Funding: This article/paper/report presents independent research funded by the National Institute for Health Research (NIHR: CDF-2012-05-126). The views expressed are those of the authors and not necessarily those of the NHS, the NIHR or the Department of Health.

Conflicts of interest: All authors declare: no financial relationships with any organisations that might have an interest in the submitted work in the previous three years; no other relationships or activities that could appear to have influenced the submitted work.

For peer review only - http://bmjopen.bmj.com/site/about/guidelines.xhtml

Data availability: The Delphi dataset is available from the lead author on reasonable request.

Ethics approval: This study had institutional ethical approval (Institute of Population Health Ethics Committee; Ref-1415-040) and was undertaken in accordance with the Tenets of Helsinki. Informed consent was obtained for the Delphi survey and from all participants in the consensus meeting.

Author contributions: FR provided oversight for the study and led the writing of the paper. FR, LH and JK contributed to data collection, reviewing the draft paper and approving the final version.

References

1. Rowe FJ, Hepworth LR, Howard C, Hanna KL, Cheyne CP, Currie J (2019) High incidence and prevalence of visual problems after acute stroke: An epidemiology study with implications for service delivery. PLoS ONE 14(3): e0213035. https://doi.org/10.1371/journal.pone.0213035
2. Hepworth LR, Rowe FJ, Walker MF, Rockliffe J, Noonan C, Howard C, Currie J. Post-stroke Visual Impairment: A Systematic Literature Review of Types and Recovery of Visual Conditions. Ophthalmology Research: An International Journal. 2015; 5(1). ISSN: 2321-7227
3. Hepworth L, Rowe FJ. Visual impairment following stroke – the impact on quality of life: a systematic review. Ophthalmology Research: an international journal. 2016; 5(2): 1-15
4. Jones SA, Shinton RA. Improving outcome in stroke patients with visual problems. Age Ageing. 2006; 35:560-5.
5. Tsai SY, Cheng CY, Hsu WM, Su TP, Liu JH, Chou P. Association between visual impairment and depression in the elderly. Journal of the Formosan Medical Association. 2003;102:86-90.
6. Chia E-M, Wang JJ, Rochtchina E, Smith W, Cumming RR, Mitchell P. Impact of bilateral visual impairment on health-related quality of life: The Blue Mountains eye study. Investigative Ophthalmology & Visual Science. 2004;45(1):71-76.
7. Pollock A, Hazelton C, Henderson CA, Angilley J, Dhillon B, Langhorne P, Livingstone K, Munro FA, Orr H, Rowe FJ, Shahani U. Interventions for visual

For peer review only - http://bmjopen.bmj.com/site/about/guidelines.xhtml

field defects in patients with stroke. Cochrane Database of Systematic Reviews 2011, Issue 10. Art. No.: CD008388. DOI: 10.1002/14651858.CD008388.pub2.

8. Hanna KL, Hepworth LR, Rowe FJ. The treatment methods for post-stroke visual impairment: a systematic review. Brain and Behaviour. 2017; 7(5): e00682. DOI: 10.1002/brb3.682
9. Bowen A, Hazelton C, Pollock A, Lincoln NB. Cognitive rehabilitation for spatial neglect following stroke. Cochrane Database of Systematic Reviews 2013, Issue 7. Art. No.: CD003586. DOI: 10.1002/14651858.CD003586.pub3
10. Hanna KL, Hepworth R, Rowe FJ. Screening methods for post stroke visual impairment; a systematic review. Disability and Rehabilitation. 2016: DOI: 10.1080/09638288.2016.1231846
11. Hepworth L, Rowe FJ, Harper R, Jarvis K, Shipman T, Rodgers H. Patient reported outcome measures for visual impairment after stroke: a systematic review. Health and Quality of Life Outcomes. 2015; DOI: 10.1186/s12955-015-0338-x
12. Williamson PR, Altman DG, Bagley H, Barnes KL, Blazeby JM, Brookes ST, Clarke M, Gargon E, Gorst S, Harman N *et al*: The COMET Handbook: version 1.0. Trials 2017, 18(3):280.
13. Prinsen CAC, Vohra S, Rose MR, Boers M, Tugwell P, Clarke M, Williamson PR, Terwee CB. How to select outcome measurement instruments for outcomes included in a 'Core Outcome Set' – a practical guideline. Trials. 2016; 17: 449. DOI: 10.1186/s13063-016-1555-2
14. COMET-initiative. Core Outcome Measures in Effectiveness Trials. http://www.comet-initiative.org/ (last accessed 4th September 2020)
15. Kirkham JJ, Davis K, Altman DG, Blazeby JM, Clarke M, Tunis S, et al. (2017) Core Outcome Set-STAndards for Development: The COS-STAD recommendations. PLoS Med 14(11): e1002447. https://doi.org/10.1371/journal.pmed.1002447
16. Kirkham JJ, Gorst S, Altman DG, Blazeby JM, Clarke M, Devane D, et al. (2016) Core Outcome Set–STAndards for Reporting: The COS-STAR Statement. PLoS Med 13(10): e1002148. https://doi.org/10.1371/journal.pmed.1002148

For peer review only - http://bmjopen.bmj.com/site/about/guidelines.xhtml

17. Rowe FJ, Hepworth LR, Kirkham JJ. Development of core outcome sets for vision screening and assessment in stroke: a Delphi and consensus study. *BMJ Open* 2019;9:e029578. doi: 10.1136/bmjopen-2019-029578
18. Pollock A, Hazelton C, Henderson CA, Angilley J, Dhillon B, Langhorne P, Livingstone K, Munro FA, Orr H, Rowe FJ, Shahani U. Interventions for age-related visual problems in patients with stroke. Cochrane Database of Systematic Reviews 2012, Issue 3. Art. No.: CD008390. DOI: 10.1002/14651858.CD008390.pub2
19. Pollock A, Hazelton C, Henderson CA, Angilley J, Dhillon B, Langhorne P, Livingstone K, Munro FA, Orr H, Rowe FJ, Shahani U. Interventions for disorders of eye movement in patients with stroke. Cochrane Database of Systematic Reviews 2011, Issue 10. Art. No.: CD008389. DOI: 10.1002/14651858.CD008389.pub2.
20. Hanna KL, Rowe FJ. Clinical versus evidence-based rehabilitation options for post-stroke visual impairment. Neuro-Ophthalmology. 2017; epub. http://dx.doi.org/10.1080/01658107.2017.1337159
21. The Vision and Stroke patient and public group (VISable). www.vision-research.co.uk (last accessed 4th September 2020)
22. www.gradeworkinggroup.org (last accessed 4th September 2020)
23. Raphael BA, Galetta KM, Jacobs DA, Markowitz CE, Liu GT, Nano-Schiavi ML, et al. Validation and test characteristics of a 10-item neuro-ophthalmic supplement to the NEI-VFQ-25. American Journal of Ophthalmology. 2006;142(6):1026–35
24. Radner W. Reading charts in ophthalmology. Graefes Archives of Clinical and Experimental Ophthalmology. 2017; 94: 714-724
25. Rowe FJ, VIS Group. Vision in Stroke cohort: profile overview of visual impairment. Brain and Behaviour. 2017*;e00771.* https://doi.org/10.1002/brb3.771
26. Rowe FJ, Conroy EJ, Bedson E, Cwiklinski E, Drummond A, García- Fiñana M, Howard C, Pollock A, Shipman T, Dodridge C, MacIntosh C, Johnson S, Noonan C, Barton G, Sackley C. A pilot randomised controlled trial comparing effectiveness of prism glasses, visual search training and standard care in hemianopia. Acta Neurologica Scandinavica. 2016; epub: DOI: 10.1111/ane.12725

For peer review only

For peer review only - http://bmjopen.bmj.com/site/about/guidelines.xhtml

27. Manny RE, Hussein M, Gwiazda J, Marsh-Tootle W, COMET study group. Repeatability of ETDRS visual acuity in children. Investigative Ophthalmology and Visual Science. 2003; 44: 3294-300
28. Stewart CE. LogMAR visual acuity measurements: limits of normality. British and Irish Orthoptic Journal. 2006; 3: 9-13
29. Negiloni K, Mazumdar D, Neog A, et al. Construction and validation of logMAR visual acuity charts in seven Indian languages. *Indian J Ophthalmol*. 2018;66(5):641-646. doi:10.4103/ijo.IJO_1165_17
30. Lam AK, Tong C, Tse J, Yu M. Repeatability of near visual acuity measurement at high and low contrast. *Clin Exp Optom*. 2008;91(5):447-452. doi:10.1111/j.1444-0938.2007.00235.x
31. Ferris FL 3rd, Bailey I. Standardizing the Measurement of Visual Acuity for Clinical Research Studies: Guidelines From the Eye Care Technology Forum. Ophthalmology. 1996 Jan;103(1):181-2.
32. Lempert P. Standardized Measurements of Visual Acuity for Clinical Research. Ophthalmology. 1996 Jul;103(7):994-5.
33. Ricci F, Cedrone C, Cerulli L. Standardized measurement of visual acuity. Ophthalmic Epidemiol. 1998 Mar;5(1):41-53.
34. Hull S, Tailor V, Balduzzi S, Rahi J, Schmucker C, Virgili G, Dahlmann-Noor A. Tests for detecting strabismus in children aged 1 to 6 years in the community. Cochrane Database of Systematic Reviews 2017, Issue 11. Art. No.: CD011221. DOI: 10.1002/14651858.CD011221.pub2.
35. Rowe FJ, Noonan CP, Manuel M. Comparison of Octopus Semi-Automated Kinetic Perimetry and Humphrey Peripheral Static Perimetry in Neuro-Ophthalmic Cases. ISRN Ophthalmology, vol. 2013, Article ID 753202. doi:10.1155/2013/753202
36. Rowe FJ, Rowlands A. Comparison of diagnostic accuracy between Octopus 900 and Goldmann kinetic visual fields. BioMed Research International. Volume 2014, Article ID 214829, doi: 10.1155/2014/214829
37. Ramirez AM, Chaya CJ, Gordon LK, Giaconi JA. (2008) A comparison of semiautomated versus manual Goldmann kinetic perimetry in patients with visually significant glaucoma. Journal of Glaucoma. 17: 111-117

For peer review only

38. Johnson CA. Standardizing the Measurement of Visual Fields for Clinical Research: Guidelines From the Eye Care Technology Forum. Ophthalmology. 1996 Jan;103(1):186-9.
39. Imaging and Perimetry Society Standards and Guidelines. Optom Vis Sci. 2011 Jan;88(1):4-7.
40. Kerr NM, Chew SS, Eady EK, Gamble GD, Danesh-Meyer HV. Diagnostic accuracy of confrontation visual field tests. Neurology. 2010 Apr 13;74(15):1184-90.
41. Holmes JM, Leske DA Hohberger GG. Defining Real Change in Prism-Cover Test Measurements. Am J Ophthalmol. 2008 Feb; 145(2): 381–385.
42. de Jongh E, Leach C, Tjon-Fo-Sang MJ, Bjerre A. Inter-examiner Variability and Agreement of the Alternate Prism Cover Test (APCT) Measurements of Strabismus Performed by 4 Examiners. Strabismus. 2014 Dec;22(4):158-66.
43. Rowe FJ. Ocular alignment and motility: moving variables in clinical trials. Trans. 34th European Strabismological Association. Bruges, Belgium. 2011, pp. 111-114
44. Rowe FJ. Measurement of manifest deviations using corneal reflections. Optometry Today, 1994 ; Nov : 36-38
45. Chen HF, Wu CY, Lin KC, Chen CL, Huang PC, Hsieh CJ, Liu JS. Rasch validation of a combined measure of basic and extended daily life functioning after stroke. Neurorehabilitation and Neural Repair. 2013; 27: 125-32
46. Radner W. Reading charts in ophthalmology/ Graefe's Archives Clinical Experimental Ophthalmology. 2017; 255: 1465-1482
47. Trauzettel-Klosinski S, Dietz K, IReST Steering Group. Standardised assessment of reading performance: the New International Reading peed Texts IReST. Investigative Ophthalmology and Vision Science. 2012; 53: 5452-61
48. Sarker SJ, Rudd AG, Douiri A, Wolfe CD. Comparison of 2 extended activities of daily living scales with the Barthel Index and predictors of their outcomes: cohort study within the South London Stroke Register (SLSR). Stroke. 2012; 43: 1362-9
49. Green J, Forster A, Young J. A test-retest reliability study of the Barthel Index, the Rivermead Mobility Index, the Nottingham Extended Activities of Daily

For peer review only - http://bmjopen.bmj.com/site/about/guidelines.xhtml

Living Scale and the Frenchay Activities Index in stroke patients. Disability and Rehabilitation. 2001; 23: 670-6

50. Legg LA, Lewis SR, Schofield-Robinson OJ, Drummond A, Langhorne P. Occupational therapy for adults with problems in activities of daily living after stroke. Cochrane Database of Systematic Reviews 2017, Issue 7. Art. No.: CD003585. DOI: 10.1002/14651858.CD003585.pub3.

51. McKenna HP. The Delphi technique: a worthwhile research approach for nursing? Journal of Advanced Nursing. 1994; 19: 1221-25

Figures

Figure 1: Flow chart of COS and COM development process

Figure 2: Core outcome sets consensus

Figure 3: Core outcome measures consensus

For peer review only

For peer review only - http://bmjopen.bmj.com/site/about/guidelines.xhtml

Table 1, COS outcome extraction from overview of reviews

COM outcomes indicated in shaded cells

**** indicates COM domains***

Outcomes for COS	COS domains
Case history – open questions	Case history – open questions
Case history – specific questions<br>Eye strain, Reading difficulty, Blurred, altered or reduced vision, Visual field loss, Awareness of full environment, Oscillopsia, Diplopia, Polyopia, Visual hallucinations, Altered colour vision, Altered movement of objects, Depth perception misjudgements, Tilted images, Distorted images, Face /object recognition, Clutter difficulty, Getting lost, Prolongation of images, Reverse image size, Glare, Visual crowding, Visual disorientation	Case history – specific questions
Case history – carer open questions	Case history – carer open questions
Case history – carer specific questions<br>Personal care issues, Eyes constantly moving/jerking, Missing things to one side, Bumping into things, Concerns over vision, Visual hallucinations, Family/friend recognition, Difficulty naming objects, Getting lost, Reading problems	Case history – carer specific questions
Case history – previous ocular history	Case history – previous ocular history
Case history – glasses wear	Case history – glasses wear
Observations – open comments	Observations – open comments
Observations – specific features<br>Lids, Pupils, Squint – misaligned eyes, Eye movements, Turning head to see, Closing one eye to see better, Misjudging distances, Wobbling eyes	Observations – specific features
logMAR charts, Snellen charts, Fixation and following observation, Vanishing optotype charts, Grating charts, Near acuity charts, Kay's pictures, Sheridan Gardiner single optotypes, Lea symbols	Visual acuity*
Fundus check, Retinal photography/OCT	Ocular health*
Cover uncover test, Alternating cover test, Observations of corneal reflections	Eye alignment position*

For peer review only - http://bmjopen.bmj.com/site/about/guidelines.xhtml

Nine positions of gaze, Horizontal gaze only, Vertical gaze only, Horizontal and vertical gaze only, Vergence, Saccade movement, Smooth pursuit movement, Optokinetic nystagmus movement, Vestibulo-ocular reflex, Hess/Lees/Harms wall charts	Eye movement assessment*
Retinal correspondence, Sensory fusion, Motor fusion, Stereopsis	Binocular vision assessment*
Prism cover test, Krimsky test, Prism reflection test, Synoptophore, Bruckner test, Maddox rod	Eye alignment measurements*
Confrontation, Static central perimetry, Static peripheral perimetry, Kinetic perimetry	Visual field assessment*
Line bisection, Cancellation task – star, balloon, heart, etc., Clock drawing, Room/environment description, Behaviour inattention test battery	Visual neglect assessment*
Observed navigation, Reading, Eye scanning, Walking observations, Activities of daily living, Self-care, Body placement, Spatial awareness, Mobility observations, Writing, Hand-eye coordination, Visual memory and cognition, Visual perception	Functional assessment*
Special test, e.g. Wilkins, iRest, Radner, Newspaper/magazine, Book	Reading assessment*
Vision-related, e.g. VFQ25, DLDV; Health-related, e.g. SF12; Activity of daily living, e.g. IADL; Extended activity of daily living, e.g. NEADL	Questionnaires*
Visual perception – checklist	Visual perception – checklist*
Swinging flashlight test	Pupil assessment*
Palpebral apertures, Lid function test	Lid assessment*
Pelli-Robson chart, Mars test, VisTech	Contrast sensitivity assessment*
Ishihara test, City test	Colour vision assessment*

OCT – Optical Coherence Tomography; iReST - International Reading Speed Test; VFQ25 – Visual Function Questionnaire 25; DLDV – Daily Living tasks Dependent on Vision; SF12 – Short Form 12; IADL – Instrumental Activities of Daily Living; NEADL – Nottingham Extended Activities of Daily Living

For peer review only - http://bmjopen.bmj.com/site/about/guidelines.xhtml

Table 2, Quality indicators of included instruments

	Visual acuity	Visual field	Ocular alignment	Ocular movement	Quality of life	Functional vision
	LogMAR	***Peripheral perimetry***	***Prism cover test***	***Rotation grading***	***VFQ-25***	***Radner reading***
Use in target population	Yes	Yes	Yes	Yes	Yes	Yes
**Content validity**						
Reliability	+	+	+	+	+	+
Responsiveness	+	+	+	+	+	+
Internal consistency	+	+	+	+	+	+
Structural validity	+	+	+	+	+	+
Measurement error	+	+	+	+	+	+
Hypothesis testing	+	+	+	+	+	+
Criterion validity	+	+	+	+	+	+
Cross-cultural validity	+	+	+	+	+	+
**Quality of evidence**	High	High	Moderate	Moderate	Moderate	Moderate
**Feasibility**						
Patient comprehension	Widely understood	Widely understood	Widely understood	Widely understood	Widely understood	Widely understood
Interpretability	Clear usage	Clear usage	Clear usage	Clear usage	Clear usage	Clear usage
Ease of administration	High	Relative	High	High	High	High
Length of instrument	Small - small number of lines	Moderate – can take time per eye	Small	Small	Moderate	Moderate
Completion time	Within minutes	15-20 minutes	Within minutes	Within minutes	Relative	Relative
Patient mental ability level	Low – use with children	Medium	Low	Low	Medium	Medium
Ease of standardisation	High ease	High	High	High	Moderate	Moderate
Clinician comprehension	Widely understood	Widely understood	Widely understood	Widely understood	Widely understood	Widely understood

For peer review only - http://bmjopen.bmj.com/site/about/guidelines.xhtml

Type of instrument	Letter chart	Perimeter	Occluder, target, prisms	Occluder, target	Questionnaire	Reading paragraphs
Cost	Low	High	Low	Low	Relative to licence	Low
Required equipment	Letter chart	Perimeter	Occluder, target, prisms	Occluder, target	Questionnaire	Book
Type of administration	Clinician-led	Clinician-led	Clinician-led	Clinician-led	Clinician-led Self-administered	Clinician-led
Availability in different settings	Yes	Yes	Yes	Yes	Yes	Yes
Copyright	Per company	Per company	Per company	Per company	Per company	Per company
Patient physical ability level	Minimal	Relative	Minimal	Minimal	Relative	Relative
Regulatory approval	No	No	No	No	No	No
Ease of score calculation	High ease	High ease	High ease	High ease	High ease	High ease

For peer review only

Table 3, Reasons for exclusion of instruments

Instrument / measurement	Exclusions
Case history	Non-standardised and subjective
Observations	Non-standardised and subjective
Visual acuity (non-logMAR options), e.g.: Snellen charts; Fixation and following observation; Vanishing optotype charts; Grating charts; Near acuity charts; Kay's pictures; Sheridan Gardiner single optotypes; Lea symbols	Inconsistencies in measurement properties
Ocular position, e.g.: Krimsky test; Prism reflection test; Synoptophore; Bruckner test; Maddox rod	Inconsistencies in measurement properties and inclusion of subjective examiner judgments
Ocular movements, e.g.: Hess/Lees/Harms wall charts	Insufficient widespread access and insufficient assessment of peripheral eye movements
Visual fields: Confrontation; Central perimetry	Subjective examiner assessment and/or insufficient assessment of the peripheral field of vision beyond 30 degrees
Reading, e.g.: IReST; Wilkins; Vocational reading charts	Reading sections set at specific font size Non-adaptable to low vision patients
Activities of daily living, e.g.: ADL (Activities of Daily Living); AMPS (Assessment of Motor and Process Skills); FAI (Frenchay Activities Index); FIM (Functional Independence Measure); MET (Multiple Errands Test); RNLI (Reintegration to Normal Living Index); SIS (Stroke Impact Scale); LIFE-H (Assessment of Life Habits); ACS (Activity Card Sort); MRS (Modified Rankin Scale)	No one specific test targeting vision-related activities of daily living
Vision-related Quality of life, e.g.: VA LV (Veterans Low Vision Visual Function Questionnaire); AI (Activity Inventory); DLTV (Daily Living Tasks Dependent on Vision); SRA-VFP (Self-Reported Assessment of Functional Visual Performance)	Inconsistencies in measurement properties

For peer review only

For peer review only - http://bmjopen.bmj.com/site/about/guidelines.xhtml

Figure 1, Flowchart of COS and COM development process

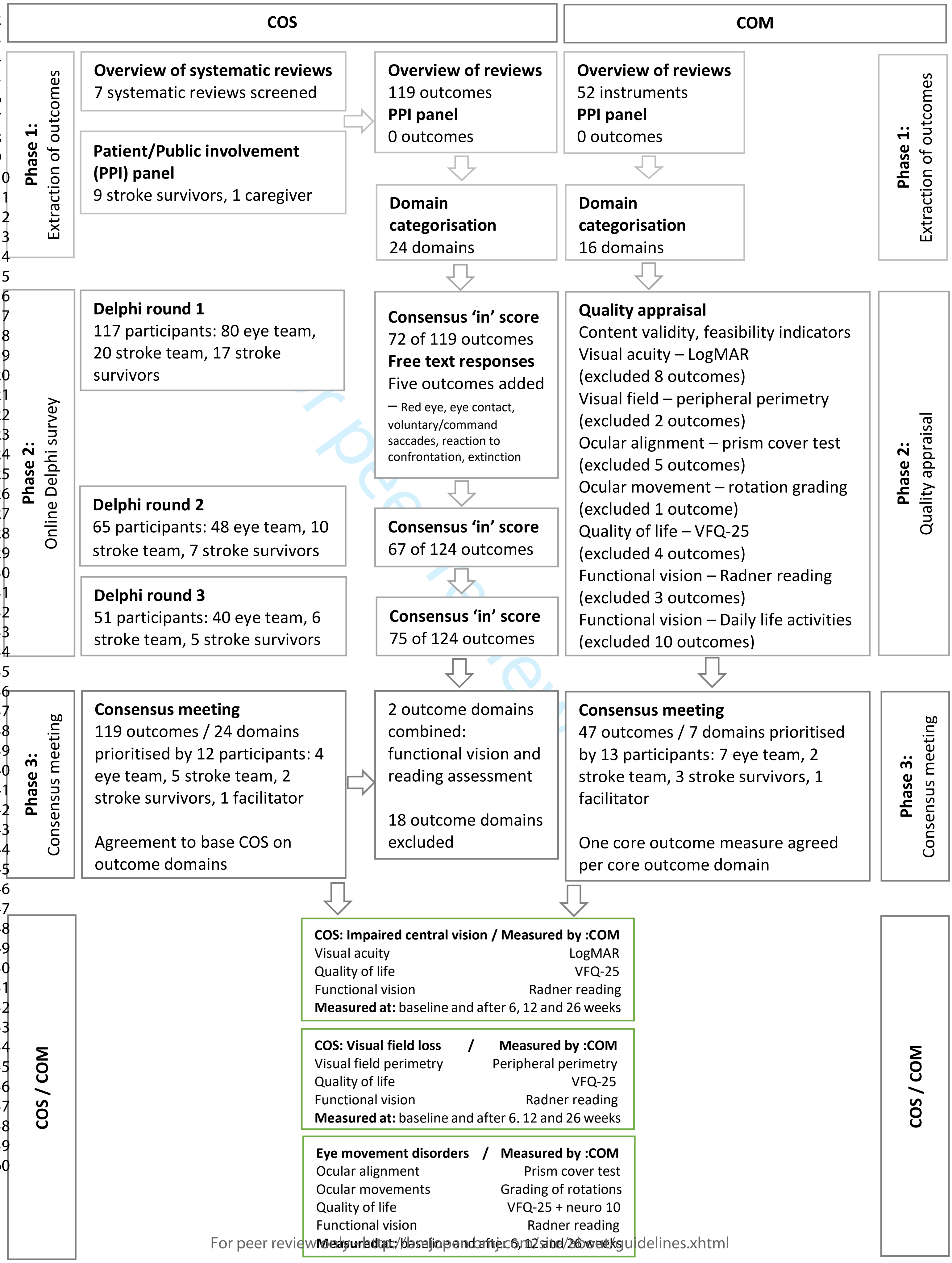

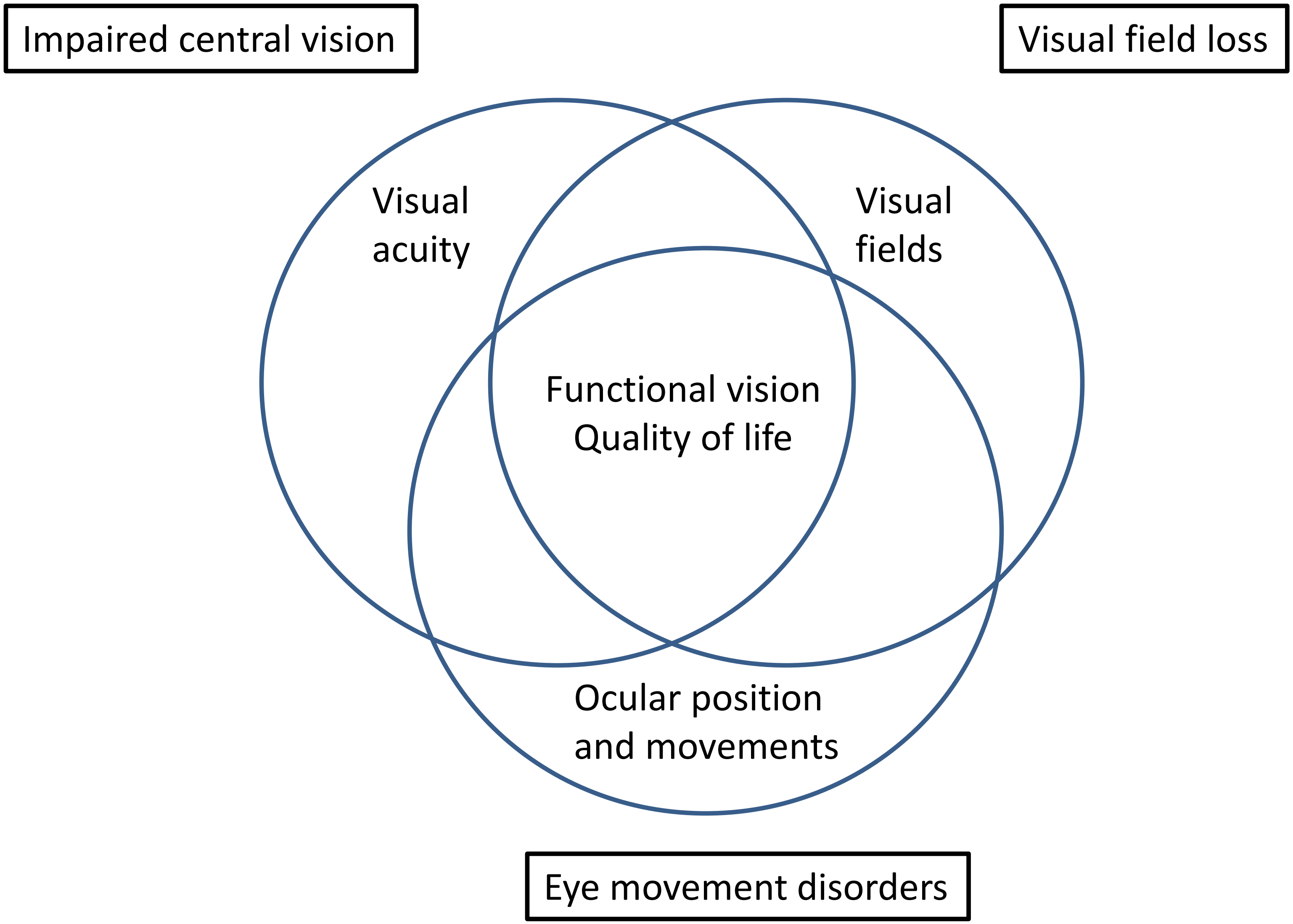
Impaired central vision
Visual field loss
Visual acuity
Visual fields
Functional vision
Quality of life
Ocular position and movements
Eye movement disorders

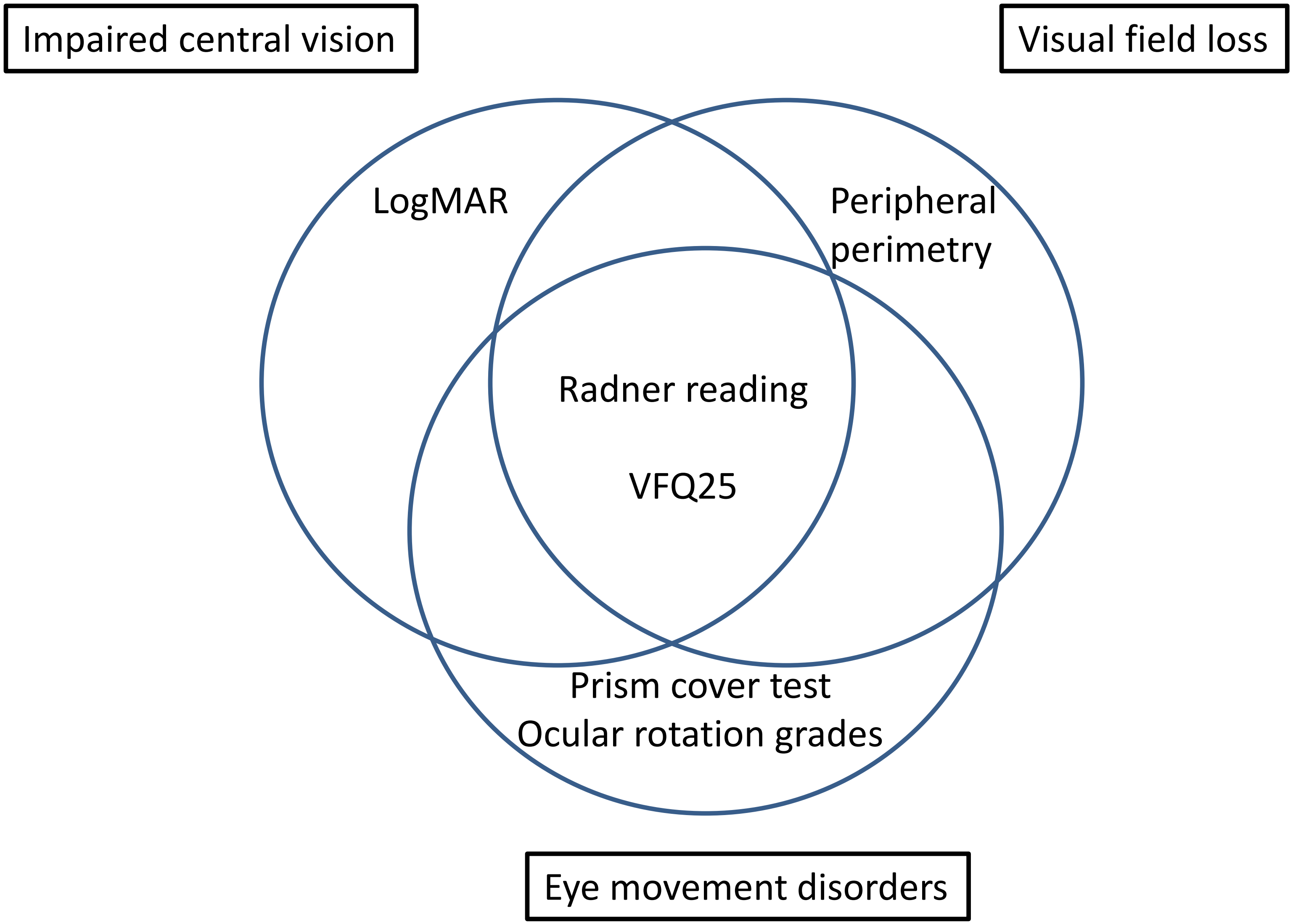
Impaired central vision
Visual field loss
LogMAR
Peripheral perimetry
Radner reading
VFQ25
Prism cover test
Ocular rotation grades
Eye movement disorders

COMET initiative registration

https://www.comet-initiative.org/Studies/Details/1420

Development of core outcome sets for vision screening and assessment in stroke: a Delphi and consensus study

OBJECTIVES: Visual impairment following stroke is common with a reported incidence of visual impairment in 60% of stroke survivors. Screening for visual impairment is neither routine nor standardised. This results in a health inequality where some stroke survivors receive comprehensive vision assessment to identify any existent visual problems while others receive no vision assessment leaving them with unmet needs from undiagnosed visual problems. The aim of this study was to define two core outcome sets (COS), one for vision screening and one for full visual assessment of stroke survivors. DESIGN: A list of potentially relevant visual assessments was created from a review of the literature. The consensus process consisted of an online 3-round Delphi survey followed by a consensus meeting of the key stakeholders. PARTICIPANTS: Stakeholders included orthoptists, occupational therapists, ophthalmologists, stroke survivors and COS users such as researchers, journal editors and guideline developers. SETTING: University. OUTCOME MEASURES: COS. RESULTS: Following the consensus process we recommend the following nine assessments for vision screening: case history, clinical observations of visual signs, visual acuity, eye alignment position, eye movement assessment, visual field assessment, visual neglect assessment, functional vision assessment and reading assessment. We recommend the following 11 assessments for full vision assessment: case history, observations, visual acuity, eye alignment position, eye movement assessment, binocular vision assessment, eye position measurement, visual field assessment, visual neglect assessment, functional vision assessment, reading assessment and quality of life questionnaires. CONCLUSIONS: COS are defined for vision screening and full vision assessment for stroke survivors. There is potential for their use in reducing heterogeneity in routine clinical practice and for improving standardisation and accuracy of vision assessment. Future research is required to evaluate the use of these COS and for further exploration of core outcome measures.

Aim

The aim of this study was to define two core outcome sets (COS), one for vision screening and one for full visual assessment of stroke survivors.

Contributors

Rowe, F. J. Hepworth, L. R. Kirkham, J. J.

Publication

Journal: BMJ Open
Volume: 9
Issue: 9
Pages: e029578 -
Year: 2019
DOI: 10.1136/bmjopen-2019-029578

Further Study Information

Current Stage: Not Applicable
Date:
Funding source(s): This article/paper/report presents independent research funded by the National Institute for Health Research (NIHR: CDF-2012-05-126).

Health Area

Disease Category: Eyes & vision, Neurology

Disease Name: Visual impairment after stroke

Target Population

Age Range: 18 - 100

Sex: Either

Nature of Intervention: Other, Screening

Stakeholders Involved

- Clinical experts
- Consumers (caregivers)
- Consumers (patients)
- Guideline developers
- Journal editors
- Researchers

Study Type

- COS for clinical trials or clinical research
- COS for practice

Method(s)

- Consensus meeting
- Delphi process
- Literature review

A list of potentially relevant visual assessments was created from a review of the literature. The consensus process consisted of an online 3-round Delphi survey followed by a consensus meeting of the key stakeholders.

only

Core Outcome Set-STandards for Reporting: The COS-STAR Statement Checklist

SECTION/TOPIC	ITEM No.	CHECKLIST ITEM	REPORTED ON PAGE NUMBER
TITLE/ABSTRACT			
Title	1a	Identify in the title that the paper reports the development of a COS	1
Abstract	1b	Provide a structured summary	2
INTRODUCTION			
Background and Objectives	2a	Describe the background and explain the rationale for developing the COS.	4
	2b	Describe the specific objectives with reference to developing a COS.	4
Scope	3a	Describe the health condition(s) and population(s) covered by the COS.	4
	3b	Describe the intervention(s) covered by the COS.	4
	3c	Describe the setting(s) in which the COS is to be applied.	4-5
METHODS			
Protocol/Registry Entry	4	Indicate where the COS development protocol can be accessed, if available, and/or the study registration details.	5
Participants	5	Describe the rationale for stakeholder groups involved in the COS development process, eligibility criteria for participants from each group, and a description of how the individuals involved were identified.	5, 6, 8, 9
Information Sources	6a	Describe the information sources used to identify an initial list of outcomes.	6, 9
	6b	Describe how outcomes were dropped/combined, with reasons (if applicable).	7, 8, 9
Consensus Process	7	Describe how the consensus process was undertaken.	8, 9
Outcome Scoring	8	Describe how outcomes were scored and how scores were summarised.	7, 8, 9
Consensus Definition	9a	Describe the consensus definition.	7, 9
	9b	Describe the procedure for determining how outcomes were included or excluded from consideration during the consensus process.	7, 8, 9
Ethics and Consent	10	Provide a statement regarding the ethics and consent issues for the study.	5, 18
RESULTS			
Protocol Deviations	11	Describe any changes from the protocol (if applicable), with reasons, and describe what impact these changes have on the results.	N/A
Participants	12	Present data on the number and relevant characteristics of the people involved at all stages of COS development.	10, 11, 12

For peer review only

Outcomes	13a	List all outcomes considered at the start of the consensus process.	10, 11, 12
	13b	Describe any new outcomes introduced and any outcomes dropped, with reasons, during the consensus process.	11, 12, 13, 14
COS	14	List the outcomes in the final COS.	13
DISCUSSION			
Limitations	15	Discuss any limitations in the COS development process.	15, 16
Conclusions	16	Provide an interpretation of the final COS in the context of other evidence, and implications for future research.	17
OTHER INFORMATION			
Funding	17	Describe sources of funding/role of funders.	17
Conflicts of Interest	18	Describe any conflicts of interest within the study team and how these were managed.	17

From: Kirkham JJ, Gorst S, Altman DG, Blazeby JM, Clarke M, Devane D, et al. (2016) Core Outcome Set–STAndards for Reporting: The COS-STAR Statement. PLoS Med 13(10): e1002148. https://doi.org/10.1371/journal.pmed.1002148

For peer review only
